# Genomic reproducibility in the bioinformatics era

**DOI:** 10.1186/s13059-024-03343-2

**Published:** 2024-08-09

**Authors:** Pelin Icer Baykal, Paweł Piotr Łabaj, Florian Markowetz, Lynn M. Schriml, Daniel J. Stekhoven, Serghei Mangul, Niko Beerenwinkel

**Affiliations:** 1 Department of Biosystems Science and Engineering, ETH Zurich, 4058 Basel, Switzerland; 2 SIB Swiss Institute of Bioinformatics, 4058 Basel, Switzerland; 3 Małopolska Centre of Biotechnology, Jagiellonian University, 30-387, Gronostajowa 7A Krakow, Poland; 4 Department of Biotechnology, Boku University Vienna, Muthgasse 18, 1190 Vienna, Austria; 5 Cancer Research UK Cambridge Research Institute, Cambridge, CB2 0RE UK; 6 Department of Oncology, University of Cambridge, Cambridge, CB2 2XZ UK; 7 Institute for Genome Sciences, University of Maryland School of Medicine, HSFIII, 670 W. Baltimore St, Baltimore, MD 21201 USA; 8 NEXUS Personalized Health Technologies, ETH Zurich, 8952 Zurich, Switzerland; 9 Titus Family Department of Clinical Pharmacy, USC Alfred E. Mann School of Pharmacy and Pharmaceutical Sciences, University of Southern California, 1540 Alcazar Street, Los Angeles, CA 90033 USA; 10 Department of Quantitative and Computational Biology, University of Southern California Dornsife College of Letters, Arts, and Sciences, Los Angeles, CA 90089 USA

**Keywords:** Reproducibility, genomics, bioinformatics tools, technical replicates, synthetic replicates

## Abstract

In biomedical research, validating a scientific discovery hinges on the reproducibility of its experimental results. However, in genomics, the definition and implementation of reproducibility remain imprecise. We argue that genomic reproducibility, defined as the ability of bioinformatics tools to maintain consistent results across technical replicates, is essential for advancing scientific knowledge and medical applications. Initially, we examine different interpretations of reproducibility in genomics to clarify terms. Subsequently, we discuss the impact of bioinformatics tools on genomic reproducibility and explore methods for evaluating these tools regarding their effectiveness in ensuring genomic reproducibility. Finally, we recommend best practices to improve genomic reproducibility.

**Supplementary Information:**

The online version contains supplementary material available at 10.1186/s13059-024-03343-2.

## Background

Reproducibility is a cornerstone principle across various scientific disciplines, each adapting the concept to suit its specific nuances [1–4]. The topic of reproducibility has garnered significant attention as experts across fields highlight the need to establish standards for validating scientific findings. Definitions of reproducibility and related concepts, such as replicability and robustness often vary by discipline. In computational research, these concepts are often defined based on whether the code and data utilized are identical. For instance, Whitaker’s matrix [5] organized the concepts of reproducibility into a framework, where the interplay between code and data determines whether the findings are reproducible, replicable, robust, or generalizable. The matrix categorizes outcomes based on the consistency of the code and data used in research. On the other hand, Essawy et al. [2] present a hierarchical pyramid model of the reproducibility taxonomy for complex computational studies, outlining the progression from repeatability, runnability, reproducibility to replicability, each requiring increasing levels of effort and time (Additional file 1: Table S1).

In genomics, reproducibility hinges on both experimental procedures and computational methods, facilitating recent strides toward precision medicine [6]. The analysis of genomic data fuels tailored treatments and improved patient outcomes. Yet, ensuring the credibility and progress of genomic medicine demands reproducible results across laboratories.

The multifaceted nature of reproducibility in genomics research is reflected in its dependence on both experimental procedures and computational methods. This complexity is underscored by the diverse steps involved in data production and analysis, spanning experimental procedures such as sample preparation and sequencing, as well as computational tasks like read alignment, variant calling, and gene expression analysis. Furthermore, the experimental variability occurring during the production of genomic data poses a considerable challenge for bioinformatics tools, as they are supposed to generate consistent genomic results under such variation.

**Fig. 1:**
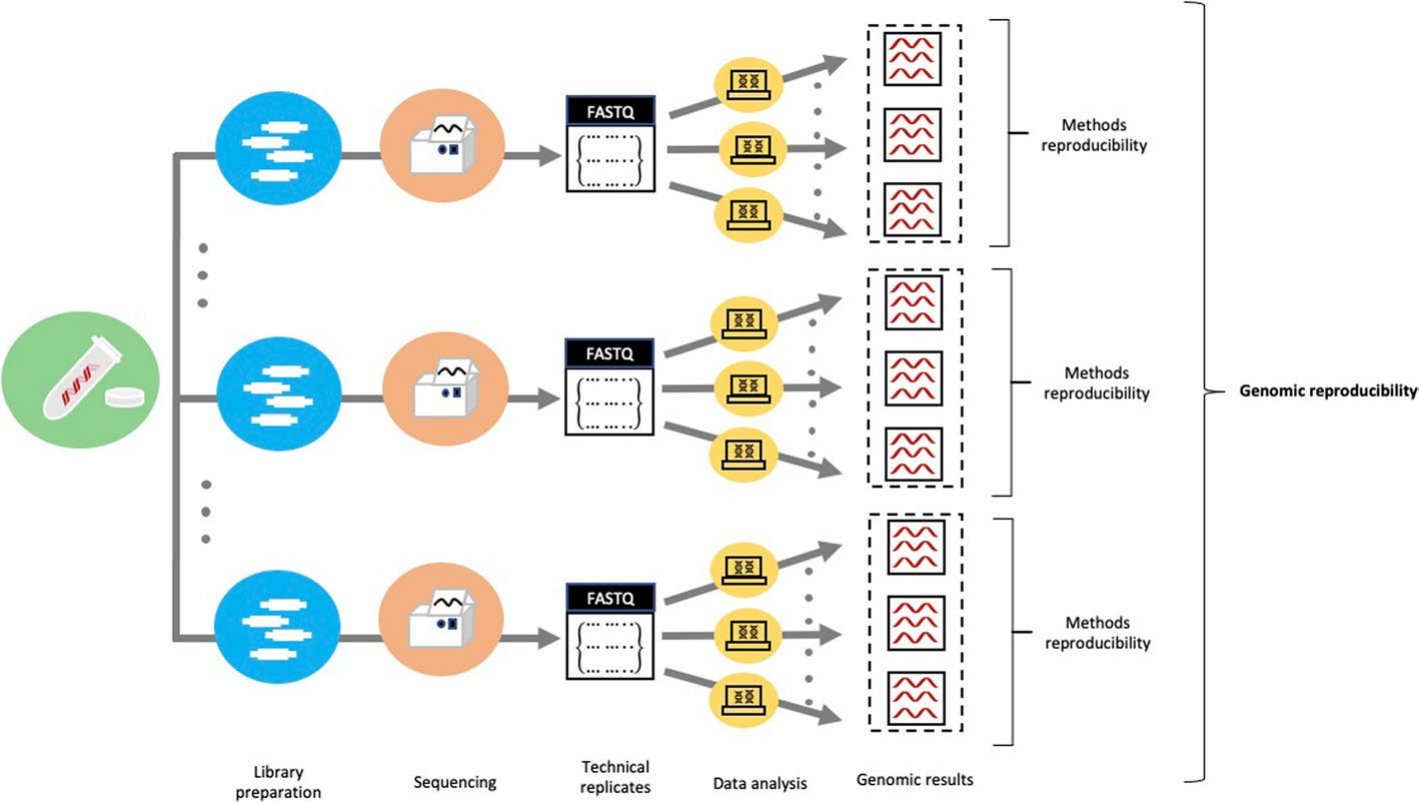
Schematic representation of three key concepts: technical replicates, methods reproducibility, and genomic reproducibility. The same sample is processed (library preparation) and sequenced multiple times, possibly in different laboratories, but using the same experimental protocols and sequencing platform. The output of these sequencing runs are technical replicates represented as FASTQ files. Data analysis is performed for each technical replicate multiple times to assess consistency of genomic results, which refers to methods reproducibility. Genomic reproducibility, on the other hand, evaluates the consistency of genomic results across technical replicates

This aspect is commonly referred to as methods reproducibility in experimental studies [4]. Methods reproducibility, as defined by Goodman et al. [4], pertains to the ability of precisely executing, to the highest degree possible, the experimental and computational procedures, using the same data and tools, in order to yield identical results [4]. In the context of genomics, methods reproducibility refers to obtaining the same results across multiple runs of the bioinformatics tools using the same parameters and genomic data (Fig. 1). Ideally, bioinformatics tools should also provide consistent results when analyzing genomic data obtained from different sequencing runs, including in different laboratories, but using the same protocols. A single, universally recognized term that describes the impact of bioinformatics tools on genomic results across such technical replicates is currently lacking. Pan et al. discuss reproducibility in the context of specific bioinformatics tasks. For instance, the reproducibility impact of read alignment tools is referred to as “aligner reproducibility,” while the reproducibility of structural variant callers is termed “caller reproducibility” [7]. The authors assess the consistency of these bioinformatics tasks across multiple tools and datasets. The closest definitions for this assessment were introduced by Goodman et al. [4] as results reproducibility and by Gundersen [8] as outcome reproducibility. Results reproducibility is the ability to obtain the same results when independent studies on different datasets are conducted with procedures closely resembling the original study [8]. However, the concept of results reproducibility was defined to target the reproduction of an experiment including a handful of statistical tests, rather than the analysis of high-dimensional and heterogeneous multi-omics data produced regularly by large collaborative genomics initiatives today. Therefore, we propose the term genomic reproducibility which measures ability to obtain consistent outcomes from bioinformatics tools using genomic data obtained from different library preparations and sequencing runs, but for fixed experimental protocols (Fig. 1).

We explore various interpretations of reproducibility before focusing on its specific application within genomics, with the goal of refining key terminology in this context. Our focus extends to the pivotal role of bioinformatics tools and their impact on genomic reproducibility, followed by an evaluation of methodologies for assessing these tools. Additionally, we examine relevant studies and technical replicate datasets as valuable resources for assessing genomic reproducibility. In conclusion, we propose actionable best practices to enhance genomic reproducibility.

## Reproducibility in genomics

Genomic reproducibility faces challenges at two pivotal junctures. The initial stage involves pre-sequencing and sequencing, where technical variability might emerge. Subsequently, during computational analysis and interpretation of genomic data, stochastic algorithms can introduce uncertainties, further impacting reproducibility. In the context of DNA sequencing, technical variability can arise from the use of diverse sequencing platforms [9] and from differences between individual flow cells [7, 10, 11]. Even if the sequencing protocol is kept identical across multiple runs, experimental variation is still expected as a result of the random sampling variance of the sequencing process and variations in library preparation [12–14]. In light of this, the objective of bioinformatics tools should be to accommodate and tolerate such experimental variation, aiming to generate consistent results across different sequencing runs and library preparations, which means achieving genomic reproducibility.

In genomics, replicates are classified into two types, biological replicates and technical replicates [15]. Biological replicates utilize multiple biological samples sharing identical conditions to quantify the inherent biological variation among them. On the other hand, technical replicates are obtained from the same biological sample sequenced multiple times, using the same experimental and computational procedures. They are used to assess and account for variability arising from the experimental process itself, such as inconsistencies in sample handling, instrument performance, or measurement techniques. Below, we focus on the best practices of using and simulating technical replicates to assess genomic reproducibility. Importantly, when assessing genomic reproducibility, we do not rely on gold standards, as the focus is not on the performance of the tools but on their capacity to maintain consistent results across technical replicates.

In practice, controlling conditions of sequencing experiments is challenging and high levels of experimental variations may compromise the ability of bioinformatics tools to maintain consistent results across technical replicates. In order to evaluate the performance of bioinformatics tools in terms of genomic reproducibility, one can consider technical replicates that specifically capture the variations among sequencing runs and library preparation techniques. This approach intentionally disregards other potential factors that could confound the results, such as sequencing protocols and platforms, allowing technical replicates acquired under the same sequencing protocols to be utilized to evaluate bioinformatics tools' impact. However, generating technical replicates can escalate both the financial burden and logistical complexity of genomic experiments. In certain cases, obtaining them may be impractical or ethically prohibitive, particularly in clinical settings.

## Bioinformatics tools can remove but also introduce unwanted variation

Bioinformatics tools play a crucial role in analyzing and eliminating undesired variation in genomic data. Variations in genomic data can arise due to multiple sources, such as experimental noise, sequencing errors, or biological artifacts. For example, homopolymer compression is employed to mitigate errors in regions with repeating nucleotide sequences by simplifying these sequences to enhance alignment accuracy [16]. Furthermore, normalization processes are used to remove batch effects or technical biases, ensuring that systematic errors do not confound the results [17]. Despite their critical roles, these tools are imperfect and can introduce various kinds of variation, both deterministic and stochastic [18].

Deterministic variations include algorithmic biases, leading alignment algorithms to favor certain sequences over others. For example, BWA [19] and Stampy [20] demonstrate a reference bias in favoring sequences containing reference alleles of a known heterozygous indel [20]. Additionally, data processing decisions such as setting a low threshold for quality filtering can include low-quality reads prone to sequencing errors, thus introducing further unwanted variation [21].

Stochastic variations in bioinformatics tools, on the other hand, stem from the intrinsic randomness of certain computational processes, such as Markov Chain Monte Carlo and genetic algorithms. Consequently, these variations may produce divergent outcomes even when identical datasets are analyzed under identical conditions.

## Genomic reproducibility of read alignment tools and variant callers

While bioinformatics tools aim to increase the accuracy of genomic data analysis and reduce sequencing errors, they can also introduce additional variation due to their built-in biases. For example, one of the challenges of read alignment tools is capturing and reporting reads mapped to repetitive regions of the reference genome, known as multi-mapped reads [22]. There exist different strategies to deal with the uncertainty of multi-mapped reads: some tools ignore these reads entirely (e.g., SNAP [23]), and others employ a deterministic approach to identify the best possible position among all the matching positions (e.g., RazerS [24] and mrFAST [25]), and finally, BWA-MEM [19] reports these multi-mapped reads with a mapping quality of zero. In the case of multi-mapping, allowing users to set a seed for a pseudo-random generator can restore the reproducibility of stochastic alignment strategies.

According to one study, random shuffling of reads affects Bowtie2 [26] and BWA-MEM [19] differently [27]. Bowtie2 is able to produce consistent alignment results irrespective of the order of the reads, while BWA-MEM [19] can show variability in results when the sequence of reads is altered. Specifically, BWA-MEM displayed variability under specific test conditions where reads were segmented and processed independently. This deviation from BWA-MEM’s typical integrated parallel processing can alter the calculated size distribution of the read inserts, as the analysis relies on smaller groups of shuffled data. This approach, although not commonly used, highlights the potential for irreproducible mapping results with BWA-MEM with respect to read order. Such variations could also influence the consistency of structural variant detection. Alkan et al. also found that structural variant calling tools produced 3.5 to 25.0% of different variant call sets with randomly shuffled data compared to the original data [27]. Furthermore, another study highlights that detecting structural variants varies significantly across different SV (structural variant) callers and even among the same callers when different read alignment tools are used [7]. It was previously shown that these variations were mainly attributed to duplications in repeat regions [27]. These studies demonstrate the potential impact of bioinformatics algorithms on the reproducibility of genomic results and emphasize the significance of assessing it with replicates.

## Opportunities to assess the impact of bioinformatics tools on genomic reproducibility

Ongoing efforts in genomics include ensuring whole-genome sequencing (WGS) reproducibility, with notable initiatives including the Genome in a Bottle (GIAB) consortium, hosted by the National Institute of Standards and Technology (NIST), and the HapMap project. The complementing efforts were performed within consecutive phases of the US FDA-led MicroArray/Sequencing Quality Control Project (MAQC/SEQC), which is helping improve microarray and next-generation sequencing technologies and foster their proper applications in discovery, development, and review of FDA-regulated products. In the MAQC-IV/SEQC phase, the aim was to assess the technical performance of next-generation sequencing platforms by generating benchmark datasets with reference samples and evaluating the advantages and limitations of various bioinformatics strategies in RNA and DNA analyses. The impact of various bioinformatics approaches on the downstream biological interpretations of RNA-seq results was comprehensively examined and the utility of RNA-seq in clinical application and safety evaluation was assessed. In SEQC2, which is the next phase of SEQC, the focus has been placed on targeted DNA- and RNA-seq to develop standard analysis protocols and quality control metrics for fit-for-purpose use of NGS data to enhance regulatory science research and precision medicine. On the other hand, consortiums such as the GIAB, and the HapMap projects provide reference materials that are used to evaluate genomic reproducibility in various studies. In Table 1, DNA and RNA-seq technical replicate datasets from major consortiums and studies are compiled, which can be used to assess genomic reproducibility.

**Table 1:** Technical replicates obtained from selected genomics consortia. The “Reference material” column indicates the reference material name used to generate technical replicates. The “Consortium” column specifies the consortium responsible for obtaining patient consent or providing the reference materials. The “Data Type” column indicates the specific type of data associated with the reference material. The “Technical replicates properties” column presents details regarding technical replicates. The “Accession” column provides information on the platform and identification numbers used to access the technical replicates. The “Link to data” column provides links to access the data associated with the technical replicates. The “Number of samples” and "Total number of base pairs" columns provide the total number of samples, the total number of technical replicates obtained from these samples, and the range of base pairs for the technical replicates, respectively. Finally, the “Study” column references the original study where these technical replicates were generated

Reference material	IDs	Consortium	Data type	Technical replicates properties	Accession	Link to data	Number of samples	Total number of base pairs	Study
Ashkenazi Jewish Trio	NIST IDs: HG002 (son) HG003 (father) HG004 (mother)	Personal Genome Project (PGP) [28]	WGS	Inter- and intra-laboratory Multiple platforms	Available in BioProject PRJNA646948 NCBI SRA: SRR12898279–SRR12898354	https://www.ncbi.nlm.nih.gov/bioproject/?term=PRJNA646948	76 samples in total, three replicates per individual	3.1 G–187.7 G	^9^
Chinese Quartet	IDs: LCL5 & LCL 6 (monozygotic twins) LCL7 & LCL8 (parents)	The Quartet Project for Quality Control and Data Integration of Multi-omics Profiling http://chinese-quartet.org	WGS	Inter- and intra-laboratory Multiple platforms	Accessible upon request	Access from^27^	9 samples in total, three replicates per individual	-	^7,27^
HapMap Trio	IDs: NA10385 NA12248 NA12249	The International HapMap Project [29]	WGS	Inter- and intra-laboratory Multiple platforms	Available in BioProject PRJNA723125	https://www.ncbi.nlm.nih.gov/bioproject/PRJNA723125	27 samples in total, three replicates per individual	128.1 G–357 G	^7^
	NA12878 (HG001) (Pilot genome)	Genome in a Bottle (GIAB) led by the NIST [30]	WGS	Inter- and intra-laboratory Multiple platforms	NCBI SRA: SRX1049768–SRX1049855	https://www.ncbi.nlm.nih.gov/sra/	88 samples in total	487.1 M–17.7 G	^7^
MAQC-I—microarrays: Universal Human RNA Reference (UHRR) and Human Brain RNA Reference (HBRR) MAQC-III (SEQC)—same samples with RNA-SEQ MAQC-IV/SEQC2—targeted RNA-Seq but also targeted DNA-Seq—UHRR plus normal male cell line (Agilent Human Reference DNA, Male, Agilent part #: 5190–8848)		MAQC-I MAQC-III MAQC-IV/SEQC2	Microarrays RNA-Seq Targeted RNA-Seq and Targeted DNA-Seq	Inter- and intra-laboratory Multiple platforms	Available in Gene Expression Omnibus (GEO) MAQC-I: GSE5350 SEQC: GSE47792 (SuperSeries) contains GSE47774 (just RNA-Seq) SEQC2: targeted RNA-Seq [not published yet] Targeted DNA-Seq	https://www.ncbi.nlm.nih.gov/geo/query/acc.cgi?acc=GSE47774 https://www.ncbi.nlm.nih.gov/bioproject/PRJNA677997	2898 samples in total 503 samples in total	100.26 M–3.98 G 538.1 M–172.3 G	^28^

Technical replicates of the Ashkenazi Trio dataset were generated to assess the performance of DNA sequencing platforms [9]. This involved generating triplicates of inter-laboratory and intra-laboratory paired-end and single-end DNA-seq samples using five Illumina and three ThermoFisher Ion Torrent platforms. This dataset can serve as a valuable resource for assessing genomic reproducibility by examining the performance of DNA-seq alignment tools and structural variant callers using both paired-end and single-end triplicate samples. The Chinese Quartet dataset, the HapMap Trio, and a pilot genome NA12878 are datasets with technical replicates that have been generated for structural variant detection studies [7, 31]. Pan et al. used technical replicates from the Chinese Quartet to assess reproducibility across three different labs using different alignment and structural variant callers [7, 31]. These technical replicates were sequenced from three different labs as triplicates representing different runs of sequencing. The same dataset was used to evaluate how sequencing centers, replicates, alignment tools, and platforms affect SV calling in NGS [31]. Additionally, The HapMap Trio and the NA12878 datasets were employed in a separate SV calling study to examine reproducibility across various factors, including sequencing platforms, labs, library preparations, alignment tools, and SV calling tools [7]. Technical replicates consist of triplicates of short-reads which can again be used to assess genomic reproducibility and the findings can be compared to the findings available in SV calling studies [7, 31]. Lastly, we mention an RNA-seq dataset provided by the SEQC consortium [32], which has been employed to assess the reproducibility of RNA-seq experiments [17, 33] and also the impact of RNA-seq data analysis tools on gene expression analysis [18]. Four samples were sequenced in 4 technical replicates each. The whole experiment was replicated in 6 different sites worldwide and another 5th replicate was created by a vendor and sent to labs for sequencing. All RNA-seq technical replicates used in these studies are made publicly available, serving as a valuable resource for assessing genomic reproducibility.

## Synthetic replicates

In certain conditions, such as when the number of technical replicates is limited for a specific type of genomic data or when reproducibility assessment requires a more controlled environment, synthetic replicates may be employed instead of technical replicates. This approach allows for a more controlled examination of the impact of specific alterations in the data. Synthetic replicates are generated in silico to mimic the variations of sequencing output expected from technical replicates. In practice, it is impossible to computationally reproduce all variations among technical replicates, but different techniques exist to generate synthetic replicates that reflect some of the variations.

**Fig. 2:**
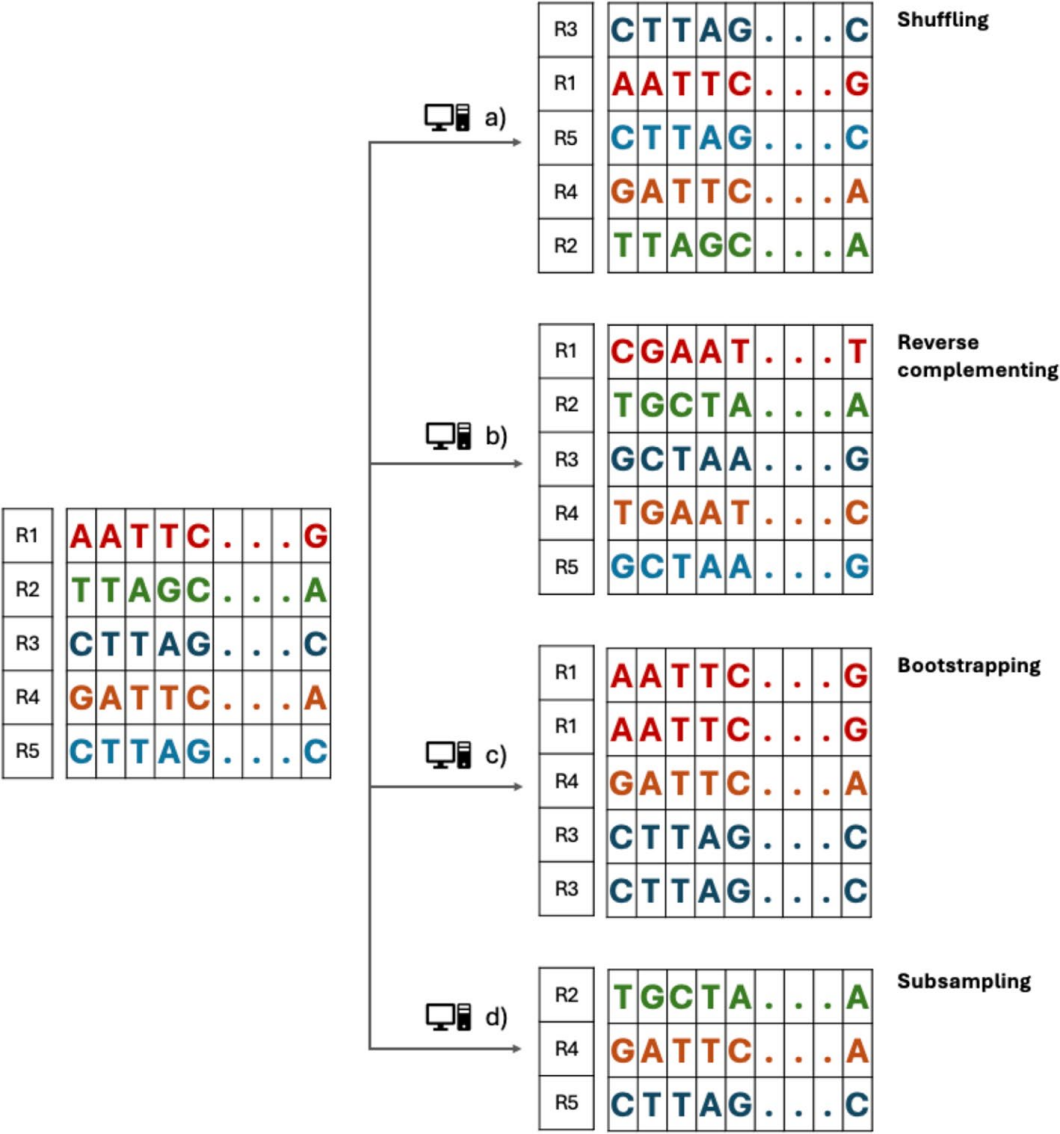
Schematic representation of generating synthetic replicates. Based on a given dataset consisting of five reads R1, …, R5 (left) four different types of synthetic replicates (right) are created by either randomly shuffling the order of the five reads (**a**), or by taking the reverse complement of each read (**b**), or by bootstrapping, i.e., resampling of the five reads with replacement (**c**), or by subsampling, i.e., selecting a subset consisting of three reads from the original five reads (**d**)

One approach to create synthetic replicates is randomly shuffling the order of the reads reported from a sequencer (Fig. 2a), which reflects the randomness of events in a sequencing experiment, such as DNA hybridization on the flow cell [27]. Another technique is to take the reverse complement of each read (Fig. 2b) to assess strand bias [34] when the reference genome is double-stranded. The bias arises due to a pronounced overabundance in one direction of NGS sequencing reads either forward or reverse, compared to the opposite direction [35]. This problem may lead to unwanted variation, which can impact genomic reproducibility. Yet another technique is bootstrapping (Fig. 2c) reflecting random sampling variance, which is a widely used type of synthetic replicate employed in many genomics, transcriptomics [36], and metagenomics [37] studies. Subsampling (Fig. 2d) is another type of synthetic replicate, which involves randomly selecting a subset of reads from the original dataset. This method simulates different levels of sequencing depth and coverage from the stochastic nature of sequencing.

Both technical replicates and synthetic replicates have their own advantages and limitations. Technical replicates contribute to a more realistic and reliable assessment by accounting for inherent variability in experimental procedures, such as different sequencing runs, and enabling rigorous statistical analysis. On the other hand, synthetic replicates offer a controlled evaluation of tools since the modifications applied to the data are known, allowing for a precise assessment against a ground truth. Hence, utilizing both types of replicates can be useful in assessing genomic reproducibility.

## Challenges in encoding and comparing structural variants

A fundamental hurdle in achieving reproducibility with structural variant callers lies in the inherent ambiguity of encoding genomic variants, stemming from biological complexities rather than technical limitations. While indels (insertions and deletions) can be left-aligned or normalized to a standard representation to facilitate comparability, complex alterations such as large deletions, insertions, duplications, inversions, and translocations present unique characteristics that complicate their consistent encoding and comparison across different replicates and bioinformatics tools. For instance, what one tool interprets as a single large deletion might be seen as multiple smaller deletions by another, due to differences in read alignments. Translocations further exemplify these difficulties, especially when they involve subtle additional changes, such as small insertions at the junction points, which might be detected by some tools but overlooked by others. These complexities significantly challenge assessing the genomic reproducibility of structural variant callers.

Moreover, the detection and characterization of SVs are intricately linked to the performance of read alignment processes. Inaccuracies or variability in aligning sequencing reads to the reference genome can have profound downstream effects on the identification and interpretation of SVs.

## Best practices to improve genomic reproducibility

We have compiled a set of recommended standards and guidelines aimed at promoting genomic reproducibility (Table 2). These recommendations are based on the expectation that bioinformatics tools already adhere to existing dependency and workflow management standards, enabling their identical execution in different settings [38]. Dependency management systems like conda, along with shared computing environments and containers such as Docker and Apptainer (formerly Singularity) [39–41] play a crucial role in ensuring consistent software environments.

**Table 2:** Recommended genomic reproducibility standards. The “Standard” column lists the names of the standards aimed at ensuring genomic reproducibility. The “Guideline” column describes the methodologies for attaining the respective standard. The columns “Essential” and “Desirable” columns categorize the levels of significance attached to each individual standard

Standard	Guideline	Essential	Optional
Documentation	- Document all the parameters of the tool, including their names, descriptions, acceptable values, and default settings - Provide detailed explanations of each parameter and its impact on the analysis or processing - Include usage examples and guidelines to help users choose appropriate parameter values - Highlight the relationship between parameter selection and reproducibility	x	
Random seeds	- Implement functionality to define random seeds for any random process involved - Document how specified random seeds impact results - Provide examples for selecting appropriate random seeds to ensure genomic reproducibility assessment	x	
Assessment of reproducibility	- Conduct a controlled experiment using synthetic replicates or technical replicates or ideally both - Report results obtained from the replicates, including any observed discrepancies or variations - Improve algorithms if needed to ensure genomic reproducibility	x	
Visualization of reproducibility performance	- Generate visual representations, such as plots and heatmaps, to examine results obtained from replicates - Clearly describe the purpose and interpretation of each visualization		x
Benchmarking	- Design reproducible benchmark studies to assess genomic reproducibility		x

We suggest the following best practices for the development and application of bioinformatics tools to ensure genomic reproducibility. First, tools should be documented sufficiently, including detailed explanations of all parameters, their default settings, usage examples, and guidelines. This documentation assists users in selecting appropriate parameter values, which is essential for reproducibility. Furthermore, tool developers should clarify the relationship between parameter selection and reproducibility in the documentation to facilitate accurate and consistent results.

The second essential requirement involves incorporating functionality that allows users to specify random seeds. By implementing this feature, developers provide users control over the random results generated by non-deterministic algorithms. This control is vital for ensuring that the same set of input data consistently produces the same output, enabling methods to assess reproducibility. This consistency is the cornerstone for enabling reliable methods to assess genomic reproducibility systematically. By setting seeds, researchers can replicate runs of bioinformatics tools under the same conditions, thereby validating the reliability of the results and facilitating a transparent evaluation of genomic reproducibility.

Another recommendation pertains to the performance assessment of the bioinformatics tool. It is essential to conduct controlled experiments using synthetic replicates, technical replicates, or a combination of both. The result obtained from these experiments, along with any observed discrepancies or variations, should be thoroughly reported. This comprehensive reporting enables researchers to evaluate the performance and reliability of the tool accurately.

Bioinformatics tool developers can enhance reproducibility by providing result visualization from replicates. However, effectively handling visualization and communicating results poses challenges due to the extensive scale and complexity of the genomic data involved. These challenges can be overcome by employing suitable visualization techniques and dimensionality reduction methods. Through careful analysis of patterns of discrepancies from the visualizations, researchers can gain valuable insights into the reliability and consistency of the results produced by the tool.

Given the vast array of bioinformatics tools and methods available, comprehensible benchmarking becomes increasingly important [40, 42]. Benchmarking can not only be used to assess performance against a ground truth, but also to assess reproducibility even in the absence of a ground truth. Reproducibility benchmarking studies are designed to evaluate the consistency of tools when used across synthetic and technical replicates. This dual approach thoroughly illuminates the reliability of tools by rigorously evaluating their performance across diverse scenarios—including variations in parameter settings and random outputs generated by different seed values. Such detailed evaluations are pivotal for pinpointing and mitigating the inherent uncertainties in parameter selection and the inherent randomness of algorithms, thereby ensuring that tools can reliably reproduce results under similar conditions.

While establishing the reliability of tools through rigorous benchmarking is vital, it's equally essential to acknowledge the potential limitations that may arise, particularly regarding the selection of cell lines for experimentation. One significant challenge is the potential presence of somatic mutations within these cell lines, which can introduce biases in evaluating tool performance. These mutations, occurring during the lifetime of the cell, can inadvertently influence experimental outcomes, leading to skewed results.

To mitigate these challenges and ensure the benchmarking studies themselves are reproducible, it is imperative that they adhere to clear guidelines. These guidelines should cover the documentation of methodologies, parameters, and experimental conditions in detail, facilitating the replication of studies by other researchers [40, 42]. Incorporating workflow management systems can further bolster the reproducibility of benchmarking studies by automating and documenting the analytical processes, thereby enhancing the consistency and transparency of genomic research.

In addition to structured benchmarking, community-driven and continuous benchmarking efforts can play important roles in advancing bioinformatics tools. For example, continuous benchmarking, as supported by Omnibenchmark [43], enables researchers to monitor tool efficacy amidst evolving datasets and computational landscapes, adapting to emerging challenges and driving progress in genomic research. This ongoing process reinforces the foundation of genomic reproducibility, promoting transparency, accountability, and adaptability within the scientific community. Embracing this structured and iterative approach to benchmarking enhances the reliability of bioinformatics tools and fortifies the foundation of genomic reproducibility.

## Conclusion

Reproducibility is critical in all fields of science, engineering, and medicine to ensure the reliability and integrity of findings and the safeness of their applications. However, there are various challenges and limitations to achieving reproducibility in practice. The field of genomics faces several hurdles to reproducibility due to rapid advancements in sequencing technologies and data generation. Each new technology introduces unique biases and sources of variation, which need to be carefully considered and addressed during data analysis. Additionally, genomic studies often involve complex bioinformatics pipelines, which are susceptible to errors and require rigorous validation.

Bioinformatics tools have made significant contributions to mitigating some of these challenges and enhancing genomic reproducibility. These tools facilitate the standardization and automation of data processing, analysis and visualizations, minimizing human error, and increasing the reliability of results. However, bioinformatics tools are not without limitations and can even introduce unwanted variations that compromise genomic reproducibility. The use of technical and synthetic replicates presents valuable approaches for evaluating essential aspects of bioinformatics algorithms and their influence on genomic reproducibility.

The use of technical replicates offers advantages, as it captures the diversity across different sequencing runs. In order to correctly assess bioinformatics tools in terms of genomic reproducibility, it is important to acknowledge that despite efforts to control experimental conditions, variations can arise due to factors such as human errors in sample preparations or unknown batch effects. These confounding factors and other experimental parameters such as variations in sequencing platforms can influence genomic results. We recommend the use of technical replicates to capture variations arising from different runs of sequencing and different library preparations.

Additionally, it is vital to understand the extent to which non-deterministic algorithms influence genomic results and to tailor the assessment of genomic reproducibility accordingly. It is important to note that while setting seeds ensures consistent results under the same conditions and facilitates reproducibility, it may also mask underlying variability across different seeds. This type of variability, if substantial, raises critical questions about genomic reproducibility.

Synthetic replicates are a fast and cost-efficient way of generating replicates in genomics. They cannot fully represent real data variation as they capture only some of the differences produced between different sequencing runs. However, they provide a useful and easily accessible way of assessing necessary features of bioinformatics algorithms and the way they impact on genomic reproducibility. When evaluating genomic reproducibility through synthetic replicates, employing shuffling and reverse complementing facilitates meaningful comparisons in read alignment. This approach enables a direct assessment of read alignments present across synthetic replicates, enhancing the effectiveness of the analysis, as the set of reads is consistent across replicates. In contrast, subsampling and bootstrapping challenge such direct comparisons; subsampling involves selecting a portion of the original reads, and bootstrapping changes the read composition by resampling with replacement. Despite this, subsampling offers valuable insights by allowing the evaluation of bioinformatics tools across different subsets of reads, serving as an indirect measure of reproducibility. Bootstrapping provides a way to simulate various sampling scenarios, creating numerous pseudo-replicates. This method enables the exploration of the inherent variability and stability in read alignment and variant detection processes under different sampling conditions. By repeatedly analyzing these varied samples, researchers can better understand how changes in read frequency and composition affect the reproducibility and accuracy of genomic analyses.

While we recommend testing tools across synthetic and technical replicates, significant concerns arise from the inherent uncertainty when using different bioinformatics tools or adjusting their settings, which leads to substantial variability in results [44]. This variability, and how method choice contributes to it, can be exploited to achieve desired outcomes, which can harm reproducibility [45] selectively. These considerations extend beyond the scope of our study but remain highly relevant and important in the broader context of genomic analysis.

Precision medicine heavily relies on accurate and reliable genomic information. However, the reliability of genomic results can only be ensured if they are reproducible by bioinformatics tools. As such, it is essential to consider reproducibility as a key evaluation criterion when assessing the quality of these tools. We recommend that both developers and users of bioinformatics tools follow the guidelines in Table 2 to ensure genomic reproducibility. By implementing these guidelines, we can improve the reliability of analyzing genomic data, and facilitate the successful translation of precision medicine to clinical practice.

### Supplementary Information

Additional file 1: Table S1: Glossary of definitions. Table containing the definitions of all terms used throughout the manuscript.

Additional file 2: Review history.

## Data Availability

No datasets or codes were used for data analysis.
