# Supplementary material for: Genomic reproducibility in the bioinformatics era
Source: Genome Biol. 2024 Aug 9;25:213. doi: 10.1186/s13059-024-03343-2 (PMC11312195; doi:10.1186/s13059-024-03343-2)
Supplement: Supplementary file 1 — Additional file 1: Table S1: Glossary of definitions. Table containing the definitions of all terms used throughout the manuscript. [file 13059_2024_3343_MOESM1_ESM.docx]

**Additional file 1: Table S1:** Glossary of definitions.

| **Term** | **Context** | **Definition** | **Reference** |
| --- | --- | --- | --- |
| Methods reproducibility | Experimental and computational | The ability to execute experimental or computational processes with absolute precision in order to yield identical results when using identical samples or data. | [^4^](https://paperpile.com/c/E6zgmQ/0Mlu0) |
| Results reproducibility | Experimental and computational | The ability to achieve consistent outcomes across independent experimental studies using different datasets, where the methods used are as closely matched to the original experiment as possible. | [^4^](https://paperpile.com/c/E6zgmQ/0Mlu0) |
| Reproducibility | Computational | The ability to produce consistent results by employing the original input data, and code. | [^5^](https://paperpile.com/c/E6zgmQ/wczm) |
| Replicability | Computational | The ability to produce similar results using the same code when the original data is replaced with similar but distinct datasets from those used in the original study. | [^5^](https://paperpile.com/c/E6zgmQ/wczm) |
| Robustness | Computational | The ability to produce consistent results when the original computational code is replaced with comparable alternatives that serve the same purpose. | [^5^](https://paperpile.com/c/E6zgmQ/wczm) |
| Generalizability | Computational | The ability to maintain core findings in an analysis that accommodates changes to both code and data. | [^5^](https://paperpile.com/c/E6zgmQ/wczm) |
| Biological replicates | Genomics | Independent samples that are derived from different biological entities of the same kind, processed under similar conditions, to assess biological variation. | [^15^](https://paperpile.com/c/E6zgmQ/WlAQ) |
| Technical replicates | Genomics | Replicates obtained from independently sequencing the same sample multiple times, possibly prepared with different library preparation techniques, but the same sequencing platform and methodology. | This study |
| Genomic reproducibility | Genomics | The ability to achieve consistent results with bioinformatics tools across technical replicates. | This study |
| Synthetic replicates | Genomics | *In silico* generated datasets to simulate technical replicates. | This study |
