# Supplementary material for: Genomic reproducibility in the bioinformatics era
Source: Genome Biol. 2024 Aug 9;25:213. doi: 10.1186/s13059-024-03343-2 (PMC11312195; doi:10.1186/s13059-024-03343-2)
Supplement: Supplementary file 2 — Additional file 2: Review history. [file 13059_2024_3343_MOESM2_ESM.docx]

Review History

**First round of review**

**Reviewer 1**

**Comments to author:**

Baykal et al. present their review/perspective of "genomic reproducibility in the bioinformatics era". Overall I think this is a very worthy topic for discussion, especially within genomics, where small biases in analysis, missed accounting of multiple tests and/or inappropriate negative controls can result in wrongly assuming some results are highly significant. Also, I was previously unaware of some of the previous work and discussion in this area. For example, the sensitivity of some tools to sequence order!

0. Number your pages please!

1. Pg ?: I did find the explanations lacking in some parts. E.g. regarding hashing tha link read attributes to read names. Why is this not deterministic?

2. Pg ?: Similarly, is there a unifying reason why permuting sequence order affects variant calls?

3. Pg ?: It seems odd not to mention cross-validation as a further option alongside the bootstrap for improving reproducibility/replicability.

4. Pg ?: Regarding "bioinformatics pipelines", a significant issue within bioinformatics is the availability and installation of tools. It is worth mentioning dependency managers (e.g. apt, CRAN, conda, ...) and shared computing environments (e.g. VMs, containers/docker, ...).

5. Pg ?: I felt the introduction lacked further context/definitions around the fundamental issues of reproducibility, replication, robustness & generalisation. IMHO the latter are where we should aim for all

research. A pyramid (e.g. Essawy et al 2020) or the more pleasing tabular "Whitaker's Matrix from Alnold et al. (2019) & Leipzig et al (2021) are reasonable visulisations of the definitions. Also, it was unclear until later in the MS why the focus was on technical and not biological replicates. This could and should be discussed earlier.

6. Pg ?: Table 1: The size of each dataset could be included too, for those researchers considering using any of these datasets.

Essawy BT, Goodall JL, Voce D, Morsy MM, Sadler JM, Choi YD, Tarboton DG, Malik T. A taxonomy for reproducible and replicable research in environmental modelling. Environmental Modelling & Software. 2020 Dec 1;134:104753.

Arnold B, Bowler L, Gibson S, Herterich P, Higman R, Krystalli A, Morley A, O'Reilly M, Whitaker K. The turing Way: a handbook for reproducible data science. Zenodo. 2019.

Leipzig J, Nüst D, Hoyt CT, Ram K, Greenberg J. The role of metadata in reproducible computational research. Patterns. 2021 Sep 10;2(9).

**Reviewer 2**

**Comments to author:**

I read this well-written paper with much interest. It handles an important issue, namely the concept of "reproducibility" in genomics. This is important, because a lot has been written about reproducibility in the last decade(s), but 1) the term is not consistently defined across fields, 2) computational biology has specificities, that the widely accepted generic definitions of reproducibility and related terms do not take into account.

The argumentation of the authors is convincing, and I think that the paper is really helpful in defining the different types of reproducibility *in the context of genomics* and providing recommendations for researchers.

Disclaimer: I am not a computational biologist, but working at the interface between biostatistics/ML, bioinformatics and metascience. It implies that I cannot assess all aspects of the paper, and that some of my comments may result from misunderstandings due to my lack of knowledge in genomics. Please feel free to ignore them in this case.

Here is a list of comments that may help to improve the manuscript.

- On p6, line 19 the term "consistent" is used in the context of experimental variation. Just after this sentence, however, the term "consistency" is used in a context where genomic data are the same. So it seems the term is used differently in these two consecutive sentences. I may be wrong... but I think that this point could be clarified.

- In Table 2, it is a bit surprising (and even contradictory) that so few recommended features are considered "desirable" (with a cross in the right column).

- p9: The topic "Stochastic data analysis algorithms are an obvious source of variation of genomic results" may better fit in the section "Bioinformatics tools can remove but also introduce unwanted variation", because it does not only concern read alignment tools and variant callers.

- I was a little confused by the structure of the paper, which is really unusual. But I understand that for an unusual paper, such a structure may be appropriate. A short paragraph at the end of the introduction to guide the reader through the paper may be useful. Such paragraphs are often terribly boring, but in the case of this paper, it may make sense.

- Since this paper is (partly) about clarifying definitions, a glossary (e.g., as supplement or in the form of a table) might be useful.

Here are some additional comments on aspects more or less related to reproducibility, that the authors may choose to incorporate/discuss or not:

- On seeds: On p10, it is stated "Allowing users to set a seed for a pseudo-random generator can, in the case of multi-mapping, restore reproducibility". It is certainly reassuring to achieve reproducibility by setting a seed, and I agree that this is important and desirable. However, I think it should be noted that this type of variability (across seeds) is undesirable if too large (even if we can control it by setting the seed). Why should we trust the results with a particular seed more than the results with another seed? There are people who argue that real reproducibility is when the results are almost identical with different seeds.

- On the robustness of tools: Should methodological researchers/software engineers who develop bioinformatics tools be (more) encouraged to incorporate features that make the results more robust to various types of technical variability?

- On benchmark studies (studies systematically comparing the behaviors of tools): They are briefly addressed at the end of the recommendations section. I think this may be done more extensively, all the more since the topic was recently highlighted in Genome Biology, see the works by Mark Robinson and team:

https://genomebiology.biomedcentral.com/articles/10.1186/s13059-019-1846-5

https://genomebiology.biomedcentral.com/articles/10.1186/s13059-019-1738-8

How could robustness of the tools be systematically assessed in benchmark studies?

How could issues such as, e.g., uncertainties regarding parameter choices or random variations (resulting from different seeds) be systematically addressed in benchmark studies?

What about the reproducibility of benchmark studies themselves?

- An issue that is slightly related to but different from genomic reproducibility is the variability of results *across tools* or across variants/parameter settings of a single tool. This variability is often huge, and opens the door to selective reporting, data dredging, fishing expeditions... At the end, we may have the situation "tell me which desired result (significant p-value, etc.) you want to get, and I will tell you which tool (variant/parameter) you have to run on your data".

In this spirit, we have recently reviewed the uncertainty related to the use of gene set analysis tools (and the resulting variability of the results across these uncertain choices):

https://wires.onlinelibrary.wiley.com/doi/10.1002/wics.1643?af=R

and how this uncertainty may be misused to enforce the desired result in a "fishing expedition" (a result that is likely unreplicable...):

https://arxiv.org/abs/2402.00754

But this may be too far away from the actual topic of the paper. Please feel free to select the aspects you find most related to your paper.

Anne-Laure Boulesteix

**Reviewer 3**

**Comments to author:**

The authors present a review of genomic reproducibility, major factors influencing it and provide guidelines for improving/ensuring such reproducibility. I like the idea to write a manuscript targeting this important question. Below are some specific comments which will hopefully lead to further improvements or comprehensiveness.

\# Comments

* bwa-mem yielding non-deterministic results: it does report a kind of random primary alignment for ambiguously aligning reads, but it also reports all other (up to n) alignments. And more importantly, it flags such alignments with a MAPQ of 0, indicating that the single primary alignment and not even the alternative alignments are not to be considered meaningful. Hence, on a qualitative level, the results of bwa-mem ARE reproducible. Just not byte by byte in the BAM/CRAM file.

* I don't understand the statement "However, some tools employ hashing methods that link read attributes to the read mapping positions" and the following sentences until the end of the paragraph. What information is associated with a hash table? Do you mean in the output or internally? If the former, where, as the attributes are put in BAM records without the need of a hash table to associate stuff. If the latter, how exactly would that affect reproducibility? I just don't get the point and the impact of this observation, sorry. Maybe it needs some more explanation or an example?

* The cited study where reshuffling led to different calling results with delly etc. is very old. I wonder if it really is the same nowadays. I get the point of the authors though that this should be checked for, but maybe they could either re-evaluate or at least mention that this is a very old finding. It is a bit unfair to delly and co to put such a statement in a current paper, especially since it will presumably be open access and at some point leak into LLMs where it will be reproduced as common knowledge. Hence, I think nowadays we should be particularly careful when replicating old and potentially outdated claims.

* Apart from random seeds, the authors could also discuss whether the underlying hardware might impact genomic reproducibility as well. Numeric instability might e.g. have an impact. I don't know whether anything like that has been reported though, it could be irrelevant. Just a thought.

* What I somehow miss in the paper is a discussion of systematic technology induced errors like homopolymer errors. What do the authors think about how those impact reproducibility? How can they be overcome (obvious answers are things like using orthogonal validation, or more direct measurements like avoiding PCR in sequencing).

* Furthermore, I would like to raise the question whether the mentioned benchmarks may also sometimes hamper the reproducibility. For example, when they rely on cell lines that are sent around, those might contain somatic variation within the cell lines. Depending on how they are evaluated, this can cause biases in the results derived from them. Another problem can occur when gold standard data is generated from consensus of multiple tools. Naturally tools behaving the same or similar to those will obtain superior performance on such benchmarks.

* Another thing I miss is the actual ambiguity in encoding genomic variants at all. indels can be left aligned to become comparable/reproducible, but for SVs this is much more difficult and in part still unsolved.

* Finally, I miss the aspect that genomics often entails the combination of tools, for which it is unavoidable to ensure general computational reproducibility that fixes the software environments and parameters of each step, as well as allowing to scale the analysis to any underlying computational infrastructure, such as it is ensured by using modern workflow management systems like Nextflow, Snakemake, Galaxy, etc. While this is of course a generic requirement, it should be mentioned in this context as well.

**Authors Response**

**Point-by-point responses to the reviewers’ comments:**

Reviewer reports:

**Reviewer #1:** Baykal et al. present their review/perspective of "genomic reproducibility in the bioinformatics era". Overall I think this is a very worthy topic for discussion, especially within genomics, where small biases in analysis, missed accounting of multiple tests and/or inappropriate negative controls can result in wrongly assuming some results are highly significant. Also, I was previously unaware of some of the previous work and discussion in this area. For example, the sensitivity of some tools to sequence order!

**Review 1 - comment 1:** Number your pages please!

***Answer:*** *We numbered the pages.*

**Review 1 - comment 2:** Pg ?: I did find the explanations lacking in some parts. E.g. regarding hashing that link read attributes to read names. Why is this not deterministic?

***Answer:*** *Thank you for the comment. Hashing is indeed deterministic. We have re-worked the text and clarified that Bowtie2 produces the same results when reads are shuffled. The new text is below:*

*“According to one study, random shuffling of reads affects Bowtie2*[*^25^*](https://paperpile.com/c/E6zgmQ/xKUWI) *and BWA-MEM*[*^18^*](https://paperpile.com/c/E6zgmQ/hmyh) *differently*[*^26^*](https://paperpile.com/c/E6zgmQ/S9iaj)*. Bowtie2 is able to produce consistent alignment results irrespective of the order of the reads, while BWA-MEM*[*^18^*](https://paperpile.com/c/E6zgmQ/hmyh) *shows variability in results when the sequence of reads is altered. This study also found that structural variant calling tools produced 3.5 to 25.0% of different variant call sets with randomly shuffled data compared to the original data*[*^26^*](https://paperpile.com/c/E6zgmQ/S9iaj)*. Furthermore, another study highlights that detecting structural variants varies significantly across different SV (structural variant) callers and even among the same callers when different read alignment tools are used*[*^7^*](https://paperpile.com/c/E6zgmQ/Plvrf)*. It was previously shown that these variations were mainly attributed to duplications in repeat regions*[*^26^*](https://paperpile.com/c/E6zgmQ/S9iaj)*. These studies demonstrate the potential impact of bioinformatics algorithms on the reproducibility of genomic results and emphasize the significance of assessing it with replicates (Table 2).”*

**Review 1 - comment 3:** Pg ?: Similarly, is there a unifying reason why permuting sequence order affects variant calls?

***Answer:*** *On page 9, we added the following sentence: “It was previously shown that these variations were mainly attributed to duplications in repeat regions*[*^26^*](https://paperpile.com/c/E6zgmQ/S9iaj)*” to describe it.*

**Review 1 - comment 4:** Pg ?: It seems odd not to mention cross-validation as a further option alongside the bootstrap for improving reproducibility/replicability.

***Answer:*** *We thank the reviewer for this important comment.* *Although cross-validation and bootstrapping both involve sampling techniques, bootstrapping specifically includes re-sampling, whereas their goals and applications differ significantly. Cross-validation is primarily used to validate the performance of a model by dividing the data into several training/validation subsets. This method tests the ability of the model to generalize unseen data by training on one subset and assessing performance on the other. In contrast, bootstrapping does not produce training/test splits of the data. Instead, it involves resampling with replacement from the original dataset to create synthetic replicates from which genomic reproducibility is assessed. In principle, subsampling can also be used to assess reproducibility.*

*We have included additional sentences that explain subsampling as a form of synthetic replicates in the "Synthetic replicates" section:*

*“Subsampling (Fig. 2d) is another type of synthetic replicate, which involves randomly selecting a subset of reads from the original dataset. This method simulates different levels of sequencing depth and coverage from the stochastic nature of sequencing. “*

*Additionally, we added several sentences to address the differences between synthetic replicates in the "Conclusion" section.*

*“When evaluating genomic reproducibility through synthetic replicates, employing shuffling and reverse complementing facilitates meaningful comparisons in read alignment. This approach enables a direct assessment of read alignments present across synthetic replicates, enhancing the effectiveness of the analysis, as the set of reads is consistent across replicates. In contrast, subsampling and bootstrapping challenge such direct comparisons; subsampling involves selecting a portion of the original reads, and bootstrapping changes the read composition by resampling with replacement. Despite this, subsampling offers valuable insights by allowing the evaluation of bioinformatics tools across different subsets of reads, serving as an indirect measure of reproducibility. Bootstrapping provides a way to simulate various sampling scenarios, creating numerous pseudo-replicates. This method enables the exploration of the inherent variability and stability in read alignment and variant detection processes under different sampling conditions. By repeatedly analyzing these varied samples, researchers can better understand how changes in read frequency and composition affect the reproducibility and accuracy of genomic analyses.”*

*We revised Figure 2 to include subsampling as a type of synthetic replicate.*

# *Figure 2: Schematic representation of generating synthetic replicates. Based on a given dataset consisting of five reads R1, R2, R3, R4, R5 (left) four different types of synthetic replicates (right) are created by either randomly shuffling the order of the five reads (a), or by taking the reverse complement of each read (b), or by bootstrapping, i.e., resampling of the five reads with replacement (c), or by subsampling, i.e., selecting a subset consisting of three reads from the original five reads (d).*

**Review 1 - comment 5:** Pg ?: Regarding "bioinformatics pipelines", a significant issue within bioinformatics is the availability and installation of tools. It is worth mentioning dependency managers (e.g. apt, CRAN, conda, ...) and shared computing environments (e.g. VMs, containers/docker, ...).

***Answer:*** *It is true that technical aspects, such as the availability of tools and the use of different computational settings impact reproducibility. In our paper, we focus on genomic reproducibility of any fixed and running data analysis pipeline, and we assume that it can be made available in an identical manner in another computational environment. However, this assumption is not always fulfilled and can be difficult to realize. On page 14, we added a sentence to acknowledge this fact and briefly point out the importance of dependency managers for achieving genomic reproducibility across computing environments.*

*“These recommendations are based on the expectation that bioinformatics tools already adhere to existing dependency and workflow management standards, enabling their identical execution in different settings*[*^34^*](https://paperpile.com/c/E6zgmQ/Gpqc)*. Dependency management systems like CRAN and conda, along with shared computing environments and containers such as Docker and Apptainer (formerly Singularity)*[*^35–37^*](https://paperpile.com/c/E6zgmQ/nZOR2+tRyT+9d8Y) *play a crucial role in ensuring consistent software environments.”*

**Review 1 - comment 6: Pg ?:** I felt the introduction lacked further context/definitions around the fundamental issues of reproducibility, replication, robustness & generalisation. IMHO the latter are where we should aim for all research. A pyramid (e.g. Essawy et al 2020) or the more pleasing tabular "Whitaker's Matrix from Alnold et al. (2019) & Leipzig et al (2021) are reasonable visulisations of the definitions. Also, it was unclear until later in the MS why the focus was on technical and not biological replicates. This could and should be discussed earlier.

***Answer:*** *Thank you for your insightful comment. We have reworked the introduction accordingly and mention the suggested contexts on page 3-4 as follows:*

*“Reproducibility is a cornerstone principle across various scientific disciplines, each adapting the concept to suit its specific nuances*[*^1–4^*](https://paperpile.com/c/E6zgmQ/qYmP+p7hD+RNxr+0Mlu0)*. The topic of reproducibility has garnered significant attention as experts across fields highlight the need to establish standards for validating scientific findings. Definitions of reproducibility and related concepts, such as replicability and robustness often vary by discipline. In computational research, these concepts are often defined based on whether the code and data utilized are identical. For instance, Whitaker's matrix*[*^5^*](https://paperpile.com/c/E6zgmQ/wczm) *organized the concepts of reproducibility into a framework, where the interplay between code and data determines whether the findings are reproducible, replicable, robust, or generalizable. The matrix categorizes outcomes based on the consistency of the code and data used in research. On the other hand, Essawy et al.*[*^2^*](https://paperpile.com/c/E6zgmQ/p7hD) *present a hierarchical pyramid model of the reproducibility taxonomy for complex computational studies, outlining the progression from repeatability, runnability, reproducibility to replicability, each requiring increasing levels of effort and time (Table S1).*

*In genomics, reproducibility hinges on both experimental procedures and computational methods, facilitating recent strides toward precision medicine*[*^6^*](https://paperpile.com/c/E6zgmQ/3dwD1)*. The analysis of genomic data fuels tailored treatments and improved patient outcomes. Yet, ensuring the credibility and progress of genomic medicine demands reproducible results across laboratories.*

*The multifaceted nature of reproducibility in genomics research is reflected in its dependence on both experimental procedures and computational methods. This complexity is underscored by the diverse steps involved in data production and analysis, spanning experimental procedures such as sample preparation and sequencing, as well as computational tasks like read alignment, variant calling, and gene expression analysis. Furthermore, the experimental variability occurring during the production of genomic data poses a considerable challenge for bioinformatics tools, as they are supposed to generate consistent genomic results under such variation.”*

*The suggested papers listed below have been cited in the edited text.*

*Essawy BT, Goodall JL, Voce D, Morsy MM, Sadler JM, Choi YD, Tarboton DG, Malik T. A taxonomy for reproducible and replicable research in environmental modelling. Environmental Modelling & Software. 2020 Dec 1;134:104753.*

*Arnold B, Bowler L, Gibson S, Herterich P, Higman R, Krystalli A, Morley A, O'Reilly M, Whitaker K. The turing Way: a handbook for reproducible data science. Zenodo. 2019.*

*Leipzig J, Nüst D, Hoyt CT, Ram K, Greenberg J. The role of metadata in reproducible computational research. Patterns. 2021 Sep 10;2(9).*

*We added the paragraph below on page 6 to define biological replicates and the reason why we focus on technical replicates rather than biological replicates.*

*“In genomics, replicates are classified into two types, biological replicates and technical replicates. Biological replicates utilize multiple biological samples sharing identical conditions to quantify the inherent biological variation among them. On the other hand, technical replicates are obtained from the same biological sample sequenced multiple times, using the same experimental and computational procedures. They are used to assess and account for variability arising from the experimental process itself, such as inconsistencies in sample handling,* *instrument performance, or measurement techniques. Below, we focus on the best practices of using and simulating technical replicates to assess genomic reproducibility.*

**Review 1 - comment 7:** Pg ?: Table 1: The size of each dataset could be included too, for those researchers considering using any of these datasets.

***Answer:*** *Table 1 is modified accordingly.*

*“Table 1: Technical replicates obtained from selected genomics consortia. The “Reference material” column indicates the reference material name used to generate technical replicates. The “Consortium” column specifies the consortium responsible for obtaining patient consent or providing the reference materials. The “Data Type” column indicates the specific type of data associated with the reference material. The “Technical replicates properties” column presents details regarding technical replicates. The “Accession” column provides information on the platform and identification numbers used to access the technical replicates. The "Link to data" column provides links to access the data associated with the technical replicates. The "Number of samples" and "Total number of base pairs" columns provide the total number of samples, the total number of technical replicates obtained from these samples, and the range of base pairs for the technical replicates, respectively. Finally, the “Study” column references the original study where these technical replicates were generated.”*

| **Reference material** | **IDs** | **Consortium** | **Data type** | **Technical replicates**  **properties** | **Accession** | **Link to data** | **Number of samples** | **Total number of base pairs** | **Study** |
| --- | --- | --- | --- | --- | --- | --- | --- | --- | --- |
| Ashkenazi Jewish Trio | NIST IDs:  HG002 (son)  HG003 (father)  HG004 (mother) | Personal Genome Project (PGP)[^42^](https://paperpile.com/c/E6zgmQ/tivLZ) | WGS | Inter- and intra-laboratory  Multiple platforms | Available in BioProject PRJNA646948  NCBI SRA:  SRR12898279–  SRR12898354 | <https://www.ncbi.nlm.nih.gov/bioproject/?term=PRJNA646948> | 76 samples in total, three replicates per individual. | 3.1 G - 187.7 G | [^9^](https://paperpile.com/c/E6zgmQ/GepU7) |
| Chinese Quartet | IDs:  LCL5 & LCL 6 (monozygotic twins)  LCL7 & LCL8 (parents) | The Quartet Project for Quality Control and Data Integration of Multi-omics Profiling  [http://chinese-quartet.org](http://chinese-quartet.org/#/dashboard) | WGS | Inter- and intra-laboratory  Multiple platforms | Accessible upon request | Access from[^27^](https://paperpile.com/c/E6zgmQ/hCBA3) | 9 samples in total, three replicates per individual. | - | [^7,27^](https://paperpile.com/c/E6zgmQ/Plvrf+hCBA3) |
| HapMap Trio | IDs:  NA10385  NA12248  NA12249 | The International HapMap Project[^43^](https://paperpile.com/c/E6zgmQ/GJvcR) | WGS | Inter- and intra-laboratory  Multiple platforms | Available in BioProject PRJNA723125 | <https://www.ncbi.nlm.nih.gov/bioproject/PRJNA723125> | 27 samples in totals, three replicates per individual. | 128.1 G - 357 G | [^7^](https://paperpile.com/c/E6zgmQ/Plvrf) |
|  | NA12878 (HG001)  (Pilot genome) | Genome in a Bottle (GIAB) led by the NIST[^44^](https://paperpile.com/c/E6zgmQ/BM9Py) | WGS | Inter- and intra-laboratory  Multiple platforms | NCBI SRA: SRX1049768 -  SRX1049855 | [https://www.ncbi.nlm.nih.gov/sra/](https://www.ncbi.nlm.nih.gov/sra/?) | 88 samples in total. | 487.1M - 17.7G | [^7^](https://paperpile.com/c/E6zgmQ/Plvrf) |
| MAQC-I - microarrays: Universal Human RNA Reference (UHRR) and Human Brain RNA Reference (HBRR)  MAQC-III (SEQC) - same samples with RNA-SEQ  MAQC-IV/SEQC2 - targeted RNA-Seq but also targeted DNA-Seq - UHRR plus  normal male cell line (Agilent Human Reference DNA, Male, Agilent part #: 5190-8848) |  | MAQC-I  MAQC-III  MAQC-IV/SEQC2 | Microarrays  RNA-Seq  Targeted RNA-Seq and Targeted DNA-Seq | Inter- and intra-laboratory  Multiple platforms | Available in Gene Expression Omnibus (GEO)  MAQC-I: GSE5350  SEQC: GSE47792 (SuperSeries) contains GSE47774 (just RNA-Seq)  SEQC2:  targeted RNA-Seq [not published yet]  Targeted DNA-Seq | <https://www.ncbi.nlm.nih.gov/geo/query/acc.cgi?acc=GSE47774>  <https://www.ncbi.nlm.nih.gov/bioproject/PRJNA677997> | 2898 samples in total.  503 samples in total. | 100.26 M - 3.98 G  538.1 M - 172.3 G | [^28^](https://paperpile.com/c/E6zgmQ/IEGoI) |

**Reviewer #2:** I read this well-written paper with much interest. It handles an important issue, namely the concept of "reproducibility" in genomics. This is important, because a lot has been written about reproducibility in the last decade(s), but 1) the term is not consistently defined across fields, 2) computational biology has specificities, that the widely accepted generic definitions of reproducibility and related terms do not take into account.

The argumentation of the authors is convincing, and I think that the paper is really helpful in defining the different types of reproducibility *in the context of genomics* and providing recommendations for researchers.

Disclaimer: I am not a computational biologist, but working at the interface between biostatistics/ML, bioinformatics and metascience. It implies that I cannot assess all aspects of the paper, and that some of my comments may result from misunderstandings due to my lack of knowledge in genomics. Please feel free to ignore them in this case.

Here is a list of comments that may help to improve the manuscript.

**Review 2 - comment 1:** On p6, line 19 the term "consistent" is used in the context of experimental variation. Just after this sentence, however, the term "consistency" is used in a context where genomic data are the same. So it seems the term is used differently in these two consecutive sentences. I may be wrong... but I think that this point could be clarified.

***Answer:*** *Thank you for the comment. We removed the first sentence starting with “Consistency” to avoid confusion.*

*“Furthermore, the experimental variability occurring during the production of genomic data poses a considerable challenge for bioinformatics tools, as they are supposed to generate consistent genomic results under such variation.*

*….*

*This aspect is commonly referred to as methods reproducibility in experimental studies*[*^4^*](https://paperpile.com/c/E6zgmQ/0Mlu0)*.”*

**Review 2 - comment 2:** In Table 2, it is a bit surprising (and even contradictory) that so few recommended features are considered "desirable" (with a cross in the right column).

*Answer: Thank you for your feedback. In this context, "desirable" was intended to mean "optional." To prevent any confusion, we've renamed the category to "optional."*

**Review 2 - comment 3:** p9: The topic "Stochastic data analysis algorithms are an obvious source of variation of genomic results" may better fit in the section "Bioinformatics tools can remove but also introduce unwanted variation", because it does not only concern read alignment tools and variant callers.

***Answer:*** *Thank you! Following your recommendation, we edited the “Bioinformatics tools can remove but also introduce unwanted variation” section and categorized unwanted variation as deterministic and stochastic variation.*

*“Bioinformatics tools play a crucial role in analyzing and eliminating undesired variation in genomic data. Variations in genomic data can arise due to multiple sources, such as experimental noise, sequencing errors, or biological artifacts. For example, homopolymer compression is employed to mitigate errors in regions with repeating nucleotide sequences by simplifying these sequences to enhance alignment accuracy*[*^15^*](https://paperpile.com/c/E6zgmQ/zadD)*. Furthermore, normalization processes are used to remove batch effects or technical biases, ensuring that systematic errors do not confound the results*[*^16^*](https://paperpile.com/c/E6zgmQ/qXEWF)*. Despite their critical roles, these tools are imperfect and can introduce various kinds of variation, both deterministic and stochastic*[*^17^*](https://paperpile.com/c/E6zgmQ/xxQFm)*.*

# *Deterministic variations include algorithmic biases, leading alignment algorithms to favor certain sequences over others. For example, BWA*[*^18^*](https://paperpile.com/c/E6zgmQ/hmyh) *and Stampy*[*^19^*](https://paperpile.com/c/E6zgmQ/Io9Pk) *demonstrate a reference bias in favoring sequences containing reference alleles of a known heterozygous indel*[*^19^*](https://paperpile.com/c/E6zgmQ/Io9Pk)*. Additionally, data processing decisions such as setting a low threshold for quality filtering can include low-quality reads prone to sequencing errors, thus introducing further unwanted variation*[*^20^*](https://paperpile.com/c/E6zgmQ/zd2Lr)*.*

*Stochastic variations in bioinformatics tools, on the other hand stem from the intrinsic randomness of certain computational processes, such as Markov Chain Monte Carlo and genetic algorithms. Consequently, these variations may produce divergent outcomes even when identical datasets are analyzed under identical conditions.”*

*We adjusted the first paragraph of the section “Genomic reproducibility of read alignment tools and variant callers” as well:*

*“While bioinformatics tools aim to increase the accuracy of genomic data analysis and reduce sequencing errors, they can also introduce additional variation due to their built-in biases. For example, one of the challenges of read alignment tools is capturing and reporting reads mapped to repetitive regions of the reference genome, known as multi-mapped reads*[*^21^*](https://paperpile.com/c/E6zgmQ/Q6kjb)*. There exist different strategies to deal with the uncertainty of multi-mapped reads: some tools ignore these reads entirely (e.g., SNAP*[*^22^*](https://paperpile.com/c/E6zgmQ/2pz6P)*), and others employ a deterministic approach to identify the best possible position among all the matching positions (e.g., RazerS*[*^23^*](https://paperpile.com/c/E6zgmQ/PWtfj) *and mrFAST*[*^24^*](https://paperpile.com/c/E6zgmQ/WOwGx)*), and finally, BWA-MEM*[*^18^*](https://paperpile.com/c/E6zgmQ/hmyh) *reports these* *multi-mapped reads with a mapping quality of zero. In the case of multi-mapping, allowing users to set a seed for a pseudo-random generator can restore the reproducibility of stochastic alignment strategies (Table 2). ”*

**Review 2 - comment 4:** I was a little confused by the structure of the paper, which is really unusual. But I understand that for an unusual paper, such a structure may be appropriate. A short paragraph at the end of the introduction to guide the reader through the paper may be useful. Such paragraphs are often terribly boring, but in the case of this paper, it may make sense.

***Answer:*** *We added a paragraph to the end of the introduction section to explain the structure of the paper.*

*“We explore various interpretations of reproducibility before focusing on its specific application within genomics, with the goal of refining key terminology in this context. Our focus extends to the pivotal role of bioinformatics tools and their impact on genomic reproducibility, followed by an evaluation of methodologies for assessing these tools. Additionally, we examine relevant studies and technical replicate datasets as valuable resources for assessing genomic reproducibility. In conclusion, we propose actionable best practices to enhance genomic reproducibility.”*

**Review 2 - comment 5:** Since this paper is (partly) about clarifying definitions, a glossary (e.g., as supplement or in the form of a table) might be useful.

***Answer:*** *We added a supplementary table as glossary to define terms used in our text.*

Table S1:

| **Term** | **Context** | **Definition** | **Reference** |
| --- | --- | --- | --- |
| Methods reproducibility | Experimental and computational | The ability to execute experimental or computational processes with absolute precision in order to yield identical results when using identical samples or data. | [^4^](https://paperpile.com/c/E6zgmQ/0Mlu0) |
| Results reproducibility | Experimental and computational | The ability to achieve consistent outcomes across independent experimental studies using different datasets, where the methods used are as closely matched to the original experiment as possible. | [^4^](https://paperpile.com/c/E6zgmQ/0Mlu0) |
| Reproducibility | Computational | The ability to produce consistent results by employing the original input data, and code. | [^5^](https://paperpile.com/c/E6zgmQ/wczm) |
| Replicability | Computational | The ability to produce similar results using the same code when the original data is replaced with similar but distinct datasets from those used in the original study. | [^5^](https://paperpile.com/c/E6zgmQ/wczm) |
| Robustness | Computational | The ability to produce consistent results when the original computational code is replaced with comparable alternatives that serve the same purpose. | [^5^](https://paperpile.com/c/E6zgmQ/wczm) |
| Generalizability | Computational | The ability to maintain core findings in an analysis that accommodates changes to both code and data. | [^5^](https://paperpile.com/c/E6zgmQ/wczm) |
| Biological replicates | Genomics | Independent samples that are derived from different biological entities of the same kind, processed under similar conditions, to assess biological variation. | [^4^](https://paperpile.com/c/E6zgmQ/WlAQ)^5^ |
| Technical replicates | Genomics | Replicates obtained from independently sequencing the same sample multiple times, possibly prepared with different library preparation techniques, but the same sequencing platform and methodology. | This study |
| Genomic reproducibility | Genomics | The ability to achieve consistent results with bioinformatics tools across technical replicates. | This study |
| Synthetic replicates | Genomics | *In silico* generated datasets to simulate technical replicates. | This study |

Here are some additional comments on aspects more or less related to reproducibility, that the authors may choose to incorporate/discuss or not:

**Review 2 - comment 6:** On seeds: On p9, it is stated “In the case of multi-mapping, allowing users to set a seed for a pseudo-random generator can restore the reproducibility of stochastic alignment strategies (Table 2).” It is certainly reassuring to achieve reproducibility by setting a seed, and I agree that this is important and desirable. However, I think it should be noted that this type of variability (across seeds) is undesirable if too large (even if we can control it by setting the seed). Why should we trust the results with a particular seed more than the results with another seed? There are people who argue that real reproducibility is when the results are almost identical with different seeds.

***Answer:*** *In our text, we claim that output control with seeds enables methods to assess reproducibility and not genomic reproducibility directly. This is explained in the following sentences:*

*“This control is vital for ensuring that the same set of input data consistently produces the same output, enabling methods to assess reproducibility. This consistency is the cornerstone for enabling reliable methods to assess genomic reproducibility systematically. By setting seeds, researchers can replicate runs of bioinformatics tools under the same conditions, thereby validating the reliability of the results and facilitating a transparent evaluation of genomic reproducibility. ”*

*We also discuss about the importance of minimal variation across seeds in our conclusion section:*

*“It is important to note that while setting seeds ensures consistent results under the same conditions and facilitates reproducibility, it may also mask underlying variability across different seeds. This type of variability, if substantial, raises critical questions about genomic reproducibility. ”*

*We also clarified in Table 2 that random seeds ensure reproducibility assessment and not reproducibility itself.*

*“Provide examples for selecting appropriate random seeds to ensure genomic reproducibility assessment.”*

**Review 2 - comment 7:** On the robustness of tools: Should methodological researchers/software engineers who develop bioinformatics tools be (more) encouraged to incorporate features that make the results more robust to various types of technical variability?

***Answer:*** *Indeed, as mentioned in Table 2, conducting an assessment of reproducibility using both synthetic and technical replicates is essential to test the algorithm and enhance it. To underscore this point, we added the following sentence to Table 2 “Improve algorithms if needed to ensure genomic reproducibility.”*

**Review 2 - comment 8:** On benchmark studies (studies systematically comparing the behaviors of tools): They are briefly addressed at the end of the recommendations section. I think this may be done more extensively, all the more since the topic was recently highlighted in Genome Biology, see the works by Mark Robinson and team:

<https://genomebiology.biomedcentral.com/articles/10.1186/s13059-019-1846-5>

<https://genomebiology.biomedcentral.com/articles/10.1186/s13059-019-1738-8>

How could robustness of the tools be systematically assessed in benchmark studies?

How could issues such as, e.g., uncertainties regarding parameter choices or random variations (resulting from different seeds) be systematically addressed in benchmark studies?

What about the reproducibility of benchmark studies themselves?

***Answer:*** *We added a final paragraph in the section “Best practices to improve genomic reproducibility section” to clarify these points:*

*“Given the vast array of bioinformatics tools and methods available, comprehensible benchmarking becomes increasingly important*[*^36,38^*](https://paperpile.com/c/E6zgmQ/BD1i+tRyT)*.. Benchmarking can not only be used to assess performance against a ground truth, but also to assess reproducibility even in the absence of a ground truth. Reproducibility benchmarking studies are designed to evaluate the consistency of tools when used across synthetic and technical replicates. This dual approach thoroughly illuminates the reliability of tools by rigorously evaluating their performance across diverse scenarios—including variations in parameter settings and random outputs generated by different seed values. Such detailed evaluations are pivotal for pinpointing and mitigating the inherent uncertainties in parameter selection and the inherent randomness of algorithms, thereby ensuring that tools can reliably reproduce results under similar conditions.*

*While establishing the reliability of tools through rigorous benchmarking is vital, it's equally essential to acknowledge the potential limitations that may arise, particularly regarding the selection of cell lines for experimentation. One significant challenge is the potential presence of somatic mutations within these cell lines, which can introduce biases in evaluating tool performance. These mutations, occurring during the lifetime of the cell, can inadvertently influence experimental outcomes, leading to skewed results.*

*To mitigate these challenges and ensure the benchmarking studies themselves are reproducible, it is imperative that they adhere to clear guidelines. These guidelines should cover the documentation of methodologies, parameters, and experimental conditions in detail, facilitating the replication of studies by other researchers*[*^36,38^*](https://paperpile.com/c/E6zgmQ/BD1i+tRyT)*. Incorporating workflow management systems can further bolster the reproducibility of benchmarking studies by automating and documenting the analytical processes, thereby enhancing the consistency and transparency of genomic research.*

*In addition to structured benchmarking, community-driven and continuous benchmarking efforts can play important roles in advancing bioinformatics tools. For example, continuous benchmarking, as supported by Omnibenchmark*[*^39^*](https://paperpile.com/c/E6zgmQ/Yhre)*, enables researchers to monitor tool efficacy amidst evolving datasets and computational landscapes, adapting to emerging challenges and driving progress in genomic research. This ongoing process reinforces the foundation of genomic reproducibility, promoting transparency, accountability, and adaptability within the scientific community. Embracing this structured and iterative approach to benchmarking enhances the reliability of bioinformatics tools and fortifies the foundation of genomic reproducibility.”*

*We also added “benchmarking” as an optional guideline in table 2.*

**Review 2 - comment 9:** An issue that is slightly related to but different from genomic reproducibility is the variability of results *across tools* or across variants/parameter settings of a single tool. This variability is often huge, and opens the door to selective reporting, data dredging, fishing expeditions... At the end, we may have the situation "tell me which desired result (significant p-value, etc.) you want to get, and I will tell you which tool (variant/parameter) you have to run on your data".

In this spirit, we have recently reviewed the uncertainty related to the use of gene set analysis tools (and the resulting variability of the results across these uncertain choices):

<https://wires.onlinelibrary.wiley.com/doi/10.1002/wics.1643?af=R>

and how this uncertainty may be misused to enforce the desired result in a "fishing expedition" (a result that is likely unreplicable...):

<https://arxiv.org/abs/2402.00754>

But this may be too far away from the actual topic of the paper. Please feel free to select the aspects you find most related to your paper.

***Answer****: Thank you for your interesting comment. Variability across tools or across different variants/parameter settings of a single tool falls outside the scope of our paper. However, we added a paragraph to discuss this issue under the conclusion section*

*“While we recommend testing tools across synthetic and technical replicates, significant concerns arise from the inherent uncertainty when using different bioinformatics tools or adjusting their settings, which leads to substantial variability in results*[*^40^*](https://paperpile.com/c/E6zgmQ/RCJp)*. This variability, and how method choice contributes to it, can be exploited to achieve desired outcomes, which can harm reproducibility*[*^41^*](https://paperpile.com/c/E6zgmQ/N6cy) *selectively. These considerations extend beyond the scope of our study but remain highly relevant and important in the broader context of genomic analysis. ”*

*Anne-Laure Boulesteix*

**Reviewer #3:** The authors present a review of genomic reproducibility, major factors influencing it and provide guidelines for improving/ensuring such reproducibility. I like the idea to write a manuscript targeting this important question. Below are some specific comments which will hopefully lead to further improvements or comprehensiveness.

\# Comments

**Review 3 - comment 1:** bwa-mem yielding non-deterministic results: it does report a kind of random primary alignment for ambiguously aligning reads, but it also reports all other (up to n) alignments. And more importantly, it flags such alignments with a MAPQ of 0, indicating that the single primary alignment and not even the alternative alignments are not to be considered meaningful. Hence, on a qualitative level, the results of bwa-mem ARE reproducible. Just not byte by byte in the BAM/CRAM file.

***Answer:*** *We corrected the sentence based on the suggestion. Below is the corrected sentence.*

*“There exist different strategies to deal with the uncertainty of multi-mapped reads: some tools ignore these reads entirely (e.g., SNAP*[*^22^*](https://paperpile.com/c/E6zgmQ/2pz6P)*), and others employ a deterministic approach to identify the best possible position among all the matching positions (e.g., RazerS*[*^23^*](https://paperpile.com/c/E6zgmQ/PWtfj) *and mrFAST*[*^24^*](https://paperpile.com/c/E6zgmQ/WOwGx)*), and finally, BWA-MEM*[*^18^*](https://paperpile.com/c/E6zgmQ/hmyh) *reports these multi-mapped reads with a mapping quality of zero.”*

**Review 3 - comment 2:** I don't understand the statement "However, some tools employ hashing methods that link read attributes to the read mapping positions" and the following sentences until the end of the paragraph. What information is associated with a hash table? Do you mean in the output or internally? If the former, where, as the attributes are put in BAM records without the need of a hash table to associate stuff. If the latter, how exactly would that affect reproducibility? I just don't get the point and the impact of this observation, sorry. Maybe it needs some more explanation or an example?

***Answer:*** *Thank you for your comment. We re-worked this paragraph.*

*“According to one study, random shuffling of reads affects Bowtie2*[*^25^*](https://paperpile.com/c/E6zgmQ/xKUWI) *and BWA-MEM*[*^18^*](https://paperpile.com/c/E6zgmQ/hmyh) *differently*[*^26^*](https://paperpile.com/c/E6zgmQ/S9iaj)*. Bowtie2 is able to produce consistent alignment results irrespective of the order of the reads, while BWA-MEM*[*^18^*](https://paperpile.com/c/E6zgmQ/hmyh) *shows variability in results when the sequence of reads is altered. This study also found that structural variant calling tools produced 3.5 to 25.0% of different variant call sets with randomly shuffled data compared to the original data*[*^26^*](https://paperpile.com/c/E6zgmQ/S9iaj)*. Furthermore, another study highlights that detecting structural variants varies significantly across different SV (structural variant) callers and even among the same callers when different read alignment tools are used*[*^7^*](https://paperpile.com/c/E6zgmQ/Plvrf)*. It was previously shown that these variations were mainly attributed to duplications in repeat regions*[*^26^*](https://paperpile.com/c/E6zgmQ/S9iaj)*. These studies demonstrate the potential impact of bioinformatics algorithms on the reproducibility of genomic results and emphasize the significance of assessing it with replicates (Table 2)..”*

**Review 3 - comment 3:** The cited study where reshuffling led to different calling results with delly etc. is very old. I wonder if it really is the same nowadays. I get the point of the authors though that this should be checked for, but maybe they could either re-evaluate or at least mention that this is a very old finding. It is a bit unfair to delly and co to put such a statement in a current paper, especially since it will presumably be open access and at some point leak into LLMs where it will be reproduced as common knowledge. Hence, I think nowadays we should be particularly careful when replicating old and potentially outdated claims.

***Answer:*** *Thank you for this important comment. This is a study from 2012. Therefore, we removed the name of the tool.*

*“According to one study, random shuffling of reads affects Bowtie2*[*^25^*](https://paperpile.com/c/E6zgmQ/xKUWI) *and BWA-MEM*[*^18^*](https://paperpile.com/c/E6zgmQ/hmyh) *differently*[*^26^*](https://paperpile.com/c/E6zgmQ/S9iaj)*. Bowtie2 is able to produce consistent alignment results irrespective of the order of the reads, while BWA-MEM*[*^18^*](https://paperpile.com/c/E6zgmQ/hmyh) *shows variability in results when the sequence of reads is altered. This study also found that structural variant calling tools* *produced 3.5 to 25.0% of different variant call sets with randomly shuffled data compared to the original data*[*^26^*](https://paperpile.com/c/E6zgmQ/S9iaj)*. Furthermore, another study highlights that detecting structural variants varies significantly across different SV (structural variant) callers and even among the same callers when different read alignment tools are used*[*^7^*](https://paperpile.com/c/E6zgmQ/Plvrf)*. It was previously shown that these variations were mainly attributed to duplications in repeat regions*[*^26^*](https://paperpile.com/c/E6zgmQ/S9iaj)*. These studies demonstrate the potential impact of bioinformatics algorithms on the reproducibility of genomic results and emphasize the significance of assessing it with replicates (Table 2).”*

**Review 3 - comment 4:** Apart from random seeds, the authors could also discuss whether the underlying hardware might impact genomic reproducibility as well. Numeric instability might e.g. have an impact. I don't know whether anything like that has been reported though, it could be irrelevant. Just a thought.

***Answer:*** *In our paper, we decided to exclude everything related to hardware and only focus on the algorithm and its implementation in the tool. Moreover, we could not find any mention of numerical instability leading to unreproducible genomics results in the literature.*

**Review 3 - comment 5:** What I somehow miss in the paper is a discussion of systematic technology induced errors like homopolymer errors. What do the authors think about how those impact reproducibility? How can they be overcome (obvious answers are things like using orthogonal validation, or more direct measurements like avoiding PCR in sequencing).

***Answer:*** *We added sentences on page 8 to elaborate about homopolymer errors. The added sentences are below.*

*“For example, homopolymer compression is employed to mitigate errors in regions with repeating nucleotide sequences by simplifying these sequences to enhance alignment accuracy*[*^15^*](https://paperpile.com/c/E6zgmQ/zadD)*”.*

**Review 3 - comment 6:** Furthermore, I would like to raise the question whether the mentioned benchmarks may also sometimes hamper the reproducibility. For example, when they rely on cell lines that are sent around, those might contain somatic variation within the cell lines. Depending on how they are evaluated, this can cause biases in the results derived from them. Another problem can occur when gold standard data is generated from consensus of multiple tools. Naturally tools behaving the same or similar to those will obtain superior performance on such benchmarks.

***Answer:*** *We added a sentence that mentions somatic mutations within cell lines as one of the limitations in assessing genomic reproducibility on page 16.*

***“****One significant challenge is the potential presence of somatic mutations within these cell lines, which can introduce biases in evaluating tool performance. These mutations, occurring during the lifetime of the cell, can inadvertently influence experimental outcomes, leading to skewed results.****”***

*Our suggested method of assessing genomic reproducibility does not incorporate gold standards, we suggest comparing results obtained from replicates. We clarified this under the “Reproducibility in genomics” section:*

*“ Importantly, when assessing genomic reproducibility, we do not rely on gold standards, as the focus is not on the performance of the tools but on their capacity to maintain consistent results across technical replicates..”*

**Review 3 - comment 7:** Another thing I miss is the actual ambiguity in encoding genomic variants at all. indels can be left aligned to become comparable/reproducible, but for SVs this is much more difficult and in part still unsolved.

***Answer:*** *We elaborated this issue on page 13, under a new section entitled “Challenges in encoding and comparing structural variants”*

*“A fundamental hurdle in achieving reproducibility with structural variant callers lies in the inherent ambiguity of encoding genomic variants, stemming from biological complexities rather than technical limitations. While indels (insertions and deletions) can be left-aligned or normalized to a standard representation to facilitate comparability, complex alterations such as large deletions, insertions, duplications, inversions, and translocations present unique characteristics that complicate their consistent encoding and comparison across different replicates and bioinformatics tools. For instance, what one tool interprets as a single large deletion might be seen as multiple smaller deletions by another, due to differences in read alignments. Translocations further exemplify these difficulties, especially when they involve subtle additional changes, such as small insertions at the junction points, which might be detected by some tools but overlooked by others. These complexities significantly challenge assessing the genomic reproducibility of structural variant callers.*

*Moreover, the detection and characterization of SVs are intricately linked to the performance of read alignment processes. Inaccuracies or variability in aligning sequencing reads to the reference genome can have profound downstream effects on the identification and interpretation of SVs.”*

**Review 3 - comment 8:** Finally, I miss the aspect that genomics often entails the combination of tools, for which it is unavoidable to ensure general computational reproducibility that fixes the software environments and parameters of each step, as well as allowing to scale the analysis to any underlying computational infrastructure, such as it is ensured by using modern workflow management systems like Nextflow, Snakemake, Galaxy, etc. While this is of course a generic requirement, it should be mentioned in this context as well.

***Answer:*** *We added a paragraph under the “Best practices to improve genomic reproducibility” to emphasize on the importance of using workflow management systems to ensure genomic reproducibility.*

*“Given the vast array of bioinformatics tools and methods available, comprehensible benchmarking becomes increasingly important*[*^36,38^*](https://paperpile.com/c/E6zgmQ/BD1i+tRyT)*. Benchmarking can not only be used to assess performance against a ground truth, but also to assess reproducibility even in the absence of a ground truth. Reproducibility benchmarking studies are designed to evaluate the consistency of tools when used across synthetic and technical replicates. This dual approach thoroughly illuminates the reliability of tools by rigorously evaluating their performance across diverse scenarios—including variations in parameter settings and random outputs generated by different seed values. Such detailed evaluations are pivotal for pinpointing and mitigating the inherent uncertainties in parameter selection and the inherent randomness of algorithms, thereby ensuring that tools can reliably reproduce results under similar conditions.*

*While establishing the reliability of tools through rigorous benchmarking is vital, it's equally essential to acknowledge the potential limitations that may arise, particularly regarding the selection of cell lines for experimentation. One significant challenge is the potential presence of somatic mutations within these cell lines, which can introduce biases in evaluating tool performance. These mutations, occurring during the lifetime of the cell, can inadvertently influence experimental outcomes, leading to skewed results.*

*To mitigate these challenges and ensure the benchmarking studies themselves are reproducible, it is imperative that they adhere to clear guidelines. These guidelines should cover the documentation of methodologies, parameters, and experimental conditions in detail, facilitating the replication of studies by other researchers*[*^36,38^*](https://paperpile.com/c/E6zgmQ/BD1i+tRyT)*. Incorporating workflow management systems can further bolster the reproducibility of benchmarking studies by automating and documenting the analytical processes, thereby enhancing the consistency and transparency of genomic research.*

*In addition to structured benchmarking, community-driven and continuous benchmarking efforts can play important roles in advancing bioinformatics tools. For example, continuous benchmarking, as supported by Omnibenchmark*[*^39^*](https://paperpile.com/c/E6zgmQ/Yhre)*, enables researchers to monitor tool efficacy amidst evolving datasets and computational landscapes, adapting to emerging challenges and driving progress in genomic research. This ongoing process reinforces the foundation of genomic reproducibility, promoting transparency, accountability, and adaptability within the scientific community. Embracing this structured and iterative approach to benchmarking enhances the reliability of bioinformatics tools and fortifies the foundation of genomic reproducibility.”*

*References*

*1.* [*Leipzig, J., Nüst, D., Hoyt, C. T., Ram, K. & Greenberg, J. The role of metadata in reproducible computational research. Patterns (N Y)* ***2****, 100322 (2021).*](http://paperpile.com/b/E6zgmQ/qYmP)

*2.* [*Bakinam T. Essawy, Jonathan L. Goodall, Daniel Voce, Mohamed M. Morsy, Jeffrey M. Sadler, Young Don Choi, David G. Tarboton, Tanu Malik. A taxonomy for reproducible and replicable research in environmental modelling. Environmental Modelling & Software* ***134****, 104753 (2020).*](http://paperpile.com/b/E6zgmQ/p7hD)

*3.* [*Arnold, B. et al. The Turing Way: A Handbook for Reproducible Data Science. doi:*](http://paperpile.com/b/E6zgmQ/RNxr)[*10.5281/zenodo.3233986*](http://dx.doi.org/10.5281/zenodo.3233986)[*.*](http://paperpile.com/b/E6zgmQ/RNxr)

*4.* [*Goodman, S. N., Fanelli, D. & Ioannidis, J. P. A. What does research reproducibility mean? Sci. Transl. Med.* ***8****, 341ps12 (2016).*](http://paperpile.com/b/E6zgmQ/0Mlu0)

*5.* [*Whitaker, K. Showing Your Working: A Guide to Reproducible Neuroimaging Analyses. (figshare, 2016). doi:*](http://paperpile.com/b/E6zgmQ/wczm)[*10.6084/M9.FIGSHARE.4244996.V1*](http://dx.doi.org/10.6084/M9.FIGSHARE.4244996.V1)[*.*](http://paperpile.com/b/E6zgmQ/wczm)

*6.* [*Hussen, B. M. et al. The emerging roles of NGS in clinical oncology and personalized medicine. Pathol. Res. Pract.****230****, 153760 (2022).*](http://paperpile.com/b/E6zgmQ/3dwD1)

*7.* [*Pan, B. et al. Assessing reproducibility of inherited variants detected with short-read whole genome sequencing. Genome Biol.* ***23****, 2 (2022).*](http://paperpile.com/b/E6zgmQ/Plvrf)

*8.* [*Erik Gundersen, O. The fundamental principles of reproducibility. Philos. Trans. A Math. Phys. Eng. Sci.* ***379****, 20200210 (2021).*](http://paperpile.com/b/E6zgmQ/8bmct)

*9.* [*Foox, J. et al. Performance assessment of DNA sequencing platforms in the ABRF Next-Generation Sequencing Study. Nat. Biotechnol.* ***39****, 1129–1140 (2021).*](http://paperpile.com/b/E6zgmQ/GepU7)

*10.* [*Website.*](http://paperpile.com/b/E6zgmQ/4xOki) *Consortium, S.-I. & SEQC/MAQC-III Consortium. A comprehensive assessment of RNA-seq accuracy, reproducibility and information content by the Sequencing Quality Control Consortium. Nature Biotechnology vol. 32 903–914 Preprint at https://doi.org/10.1038/nbt.2957 (2014).*

*11.* [*Website.*](http://paperpile.com/b/E6zgmQ/hWrL1) *Blainey, P., Krzywinski, M. & Altman, N. Replication. Nature Methods vol. 11 879–880 Preprint at https://doi.org/10.1038/nmeth.3091 (2014).*

*12.* [*Marioni, J. C., Mason, C. E., Mane, S. M., Stephens, M. & Gilad, Y. RNA-seq: an assessment of technical reproducibility and comparison with gene expression arrays. Genome Res.* ***18****, 1509–1517 (2008).*](http://paperpile.com/b/E6zgmQ/ak1BT)

*13.* [*Łabaj, P. P. et al. Characterization and improvement of RNA-Seq precision in quantitative transcript expression profiling. Bioinformatics* ***27****, i383–i391 (2011).*](http://paperpile.com/b/E6zgmQ/k0VHt)

*14.* [*Fu, G. K. et al. Molecular indexing enables quantitative targeted RNA sequencing and reveals poor efficiencies in standard library preparations. Proc. Natl. Acad. Sci. U. S. A.* ***111****, 1891–1896 (2014).*](http://paperpile.com/b/E6zgmQ/bCgFh)

*15.* [*Mapping-friendly sequence reductions: Going beyond homopolymer compression. iScience* ***25****, 105305 (2022).*](http://paperpile.com/b/E6zgmQ/zadD)

*16.* [*Li, S. et al. Detecting and correcting systematic variation in large-scale RNA sequencing data. Nat. Biotechnol.* ***32****, 888–895 (2014).*](http://paperpile.com/b/E6zgmQ/qXEWF)

*17.* [*Tong, L. et al. Impact of RNA-seq data analysis algorithms on gene expression estimation and downstream prediction. Sci. Rep.* ***10****, 17925 (2020).*](http://paperpile.com/b/E6zgmQ/xxQFm)

*18.* [*Li, H. Aligning sequence reads, clone sequences and assembly contigs with BWA-MEM. arXiv preprint arXiv:1303. 3997.*](http://paperpile.com/b/E6zgmQ/hmyh)

*19.* [*Lunter, G. & Goodson, M. Stampy: a statistical algorithm for sensitive and fast mapping of Illumina sequence reads. Genome Res.* ***21****, 936–939 (2011).*](http://paperpile.com/b/E6zgmQ/Io9Pk)

*20.* [*Ros-Freixedes, R. et al. Impact of index hopping and bias towards the reference allele on accuracy of genotype calls from low-coverage sequencing. Genet. Sel. Evol.* ***50****, 64 (2018).*](http://paperpile.com/b/E6zgmQ/zd2Lr)

*21.* [*Alser, M. et al. Technology dictates algorithms: recent developments in read alignment. Genome Biol.* ***22****, 249 (2021).*](http://paperpile.com/b/E6zgmQ/Q6kjb)

*22.* [*Zaharia, M. et al. Faster and more accurate sequence alignment with SNAP. arXiv preprint arXiv:1111. 5572.*](http://paperpile.com/b/E6zgmQ/2pz6P)

*23.* [*Weese, D., Holtgrewe, M. & Reinert, K. RazerS 3: faster, fully sensitive read mapping. Bioinformatics* ***28****, 2592–2599 (2012).*](http://paperpile.com/b/E6zgmQ/PWtfj)

*24.* [*Alkan, C. et al. Personalized copy number and segmental duplication maps using next-generation sequencing. Nat. Genet.* ***41****, 1061–1067 (2009).*](http://paperpile.com/b/E6zgmQ/WOwGx)

*25.* [*Langmead, B., Trapnell, C., Pop, M. & Salzberg, S. L. Ultrafast and memory-efficient alignment of short DNA sequences to the human genome. Genome Biology vol. 10 R25 Preprint at https://doi.org/*](http://paperpile.com/b/E6zgmQ/xKUWI)[*10.1186/gb-2009-10-3-r25*](http://dx.doi.org/10.1186/gb-2009-10-3-r25)[*(2009).*](http://paperpile.com/b/E6zgmQ/xKUWI)

*26.* [*Firtina, C. & Alkan, C. On genomic repeats and reproducibility. Bioinformatics vol. 32 2243–2247 Preprint at https://doi.org/*](http://paperpile.com/b/E6zgmQ/S9iaj)[*10.1093/bioinformatics/btw139*](http://dx.doi.org/10.1093/bioinformatics/btw139) [*(2016).*](http://paperpile.com/b/E6zgmQ/S9iaj)

*27.* [*Khayat, M. M. et al. Hidden biases in germline structural variant detection. Genome Biol.* ***22****, 347 (2021).*](http://paperpile.com/b/E6zgmQ/hCBA3)

*28.* [*Consortium, S.-I. & SEQC/MAQC-III Consortium. A comprehensive assessment of RNA-seq accuracy, reproducibility and information content by the Sequencing Quality Control Consortium. Nature Biotechnology vol. 32 903–914 Preprint at https://doi.org/*](http://paperpile.com/b/E6zgmQ/IEGoI)[*10.1038/nbt.2957*](http://dx.doi.org/10.1038/nbt.2957) [*(2014).*](http://paperpile.com/b/E6zgmQ/IEGoI)

*29.* [*Munro, S. A. et al. Assessing technical performance in differential gene expression experiments with external spike-in RNA control ratio mixtures. Nat. Commun.* ***5****, 5125 (2014).*](http://paperpile.com/b/E6zgmQ/AfDiL)

*30.* [*Guo, Y. et al. The effect of strand bias in Illumina short-read sequencing data. BMC Genomics* ***13****, 666 (2012).*](http://paperpile.com/b/E6zgmQ/1rmZt)

*31.* [*Validation of a Customized Bioinformatics Pipeline for a Clinical Next-Generation Sequencing Test Targeting Solid Tumor–Associated Variants. J. Mol. Diagn.* ***20****, 355–365 (2018).*](http://paperpile.com/b/E6zgmQ/eHvWH)

*32.* [*Al Seesi, S., Tiagueu, Y. T., Zelikovsky, A. & Măndoiu, I. I. Bootstrap-based differential gene expression analysis for RNA-Seq data with and without replicates. BMC Genomics* ***15 Suppl 8****, S2 (2014).*](http://paperpile.com/b/E6zgmQ/YYWlU)

*33.* [*Saremi, B., Kohls, M., Liebig, P., Siebert, U. & Jung, K. Measuring reproducibility of virus metagenomics analyses using bootstrap samples from FASTQ-files. Bioinformatics* ***37****, 1068–1075 (2021).*](http://paperpile.com/b/E6zgmQ/dhzgE)

*34.* [*Alser, M. et al. Packaging and containerization of computational methods. Nat. Protoc. (2024) doi:*](http://paperpile.com/b/E6zgmQ/Gpqc)[*10.1038/s41596-024-00986-0*](http://dx.doi.org/10.1038/s41596-024-00986-0)[*.*](http://paperpile.com/b/E6zgmQ/Gpqc)

*35.* [*Brito, J. J. et al. Recommendations to enhance rigor and reproducibility in biomedical research. Gigascience* ***9****, (2020).*](http://paperpile.com/b/E6zgmQ/nZOR2)

*36.* [*Weber, L. M. et al. Essential guidelines for computational method benchmarking. Genome Biol.* ***20****, 1–12 (2019).*](http://paperpile.com/b/E6zgmQ/tRyT)

*37.* [*Mangul, S. et al. Challenges and recommendations to improve the installability and archival stability of omics computational tools. PLoS Biol.* ***17****, e3000333 (2019).*](http://paperpile.com/b/E6zgmQ/9d8Y)

*38.* [*Mangul, S. et al. Systematic benchmarking of omics computational tools. Nat. Commun.* ***10****, 1393 (2019).*](http://paperpile.com/b/E6zgmQ/BD1i)

*39.* [*Home - OMNIBENCHMARK.*](http://paperpile.com/b/E6zgmQ/Yhre) [*https://omnibenchmark.org*](https://omnibenchmark.org/)[*.*](http://paperpile.com/b/E6zgmQ/Yhre)

*40.* [*Wünsch, M., Sauer, C., Callahan, P., Hinske, L. C. & Boulesteix, A.-L. From RNA sequencing measurements to the final results: A practical guide to navigating the choices and uncertainties of gene set analysis. Wiley Interdiscip. Rev. Comput. Stat.* ***16****, e1643 (2024).*](http://paperpile.com/b/E6zgmQ/RCJp)

*41.* [*Wünsch, M., Sauer, C., Herrmann, M., Hinske, L. C. & Boulesteix, A.-L. To tweak or not to tweak. How exploiting flexibilities in gene set analysis leads to over-optimism. (2024).*](http://paperpile.com/b/E6zgmQ/N6cy)

*42.* [*Ball, M. P. et al. A public resource facilitating clinical use of genomes. Proc. Natl. Acad. Sci. U. S. A.* ***109****, 11920–11927 (2012).*](http://paperpile.com/b/E6zgmQ/tivLZ)

*43.* [*Consortium, †the International Hapmap & †The International HapMap Consortium. The International HapMap Project. Nature vol. 426 789–796 Preprint at https://doi.org/*](http://paperpile.com/b/E6zgmQ/GJvcR)[*10.1038/nature02168*](http://dx.doi.org/10.1038/nature02168) [*(2003).*](http://paperpile.com/b/E6zgmQ/GJvcR)

*44.* [*Zook, J. M. et al. An open resource for accurately benchmarking small variant and reference calls. Nat. Biotechnol.****37****, 561–566 (2019).*](http://paperpile.com/b/E6zgmQ/BM9Py)

*45.* [*Bell, G. Replicates and repeats. BMC Biol.* ***14****, 28 (2016).*](http://paperpile.com/b/E6zgmQ/WlAQ)

**Second round of review**

**Reviewer 2**

The authors nicely addressed my concerns.

**Reviewer 3**

Thanks for the detailed responses and improvements in the manuscript. From my side, only two comments remain.

\# Comments
1. Referring to Review 3 - comment 2: I have checked the cited manuscript (reference 26) that reports on BWA-MEM being non-deterministic. They chunked the input fastqs and ran BWA-MEM in a scatter-gather way on them. This is extremely unusual and artificial, since BWA-MEM has internal parallelization. I think the non-determinism in this special scenario might stem from the fact that BWA-MEM estimates the insert size distribution from the given input reads. When chunking, you get smaller subsets and when shuffling the reads before chunking you get potentially differences in the insert size estimates. All in all, a very special and unrealistic scenario. It can be pointed out here, but I would highly recommend to mention that BWA-MEM was used in an unusual way to achieve the non-determinism.
2. Page 14: CRAN is not a dependency mangement system. It is a package archive.

**Authors Response**

**Point-by-point responses to the reviewers’ comments:**

Reviewer #2: The authors nicely addressed my concerns.

*Answer: Thank you very much.*

Reviewer #3: Thanks for the detailed responses and improvements in the manuscript. From my side, only two comments remain.

\# Comments
1. Referring to Review 3 - comment 2: I have checked the cited manuscript (reference 26) that reports on BWA-MEM being non-deterministic. They chunked the input fastqs and ran BWA-MEM in a scatter-gather way on them. This is extremely unusual and artificial, since BWA-MEM has internal parallelization. I think the non-determinism in this special scenario might stem from the fact that BWA-MEM estimates the insert size distribution from the given input reads. When chunking, you get smaller subsets and when shuffling the reads before chunking you get potentially differences in the insert size estimates. All in all, a very special and unrealistic scenario. It can be pointed out here, but I would highly recommend to mention that BWA-MEM was used in an unusual way to achieve the non-determinism.
*Answer: Thank you very much for this comment. Below is the corrected paragraph.*

*“According to one study, random shuffling of reads affects Bowtie2[25] and BWA-MEM18 differently[26]. Bowtie2 is able to produce consistent alignment results irrespective of the order of the reads, while BWA-MEM[18] can show variability in results when the sequence of reads is altered. Specifically, BWA-MEM displayed variability under specific test conditions where reads were segmented and processed independently. This deviation from BWA-MEM’s typical integrated parallel processing can alter the calculated size distribution of the read inserts, as the analysis relies on smaller groups of shuffled data. This approach, although not commonly used, highlights the potential for irreproducible mapping results with BWA-MEM with respect to read order. Such variations could also influence the consistency of structural variant detection. Can Alkan et al. also found that structural variant calling tools produced 3.5 to 25.0% of different variant call sets with randomly shuffled data compared to the original data[26]. Furthermore, another study highlights that detecting structural variants varies significantly across different SV (structural variant) callers and even among the same callers when different read alignment tools are used[7]. It was previously shown that these variations were mainly attributed to duplications in repeat regions[26]. These studies demonstrate the potential impact of bioinformatics algorithms on the reproducibility of genomic results and emphasize the significance of assessing it with replicates (Table 2).”*

2. Page 14: CRAN is not a dependency mangement system. It is a package archive.

*Answer: Thank you for the comment. We removed CRAN from the corresponding sentence.

“Dependency management systems like conda, along with shared computing environments and containers such as Docker and Apptainer (formerly Singularity)[35–37] play a crucial role in ensuring consistent software environments.”*
